# The DNA/RNA autophagy protein SIDT2 as a novel neuropathological hallmark in Huntington disease

**DOI:** 10.1111/bpa.70088

**Published:** 2026-02-24

**Authors:** Sanaz Gabery, Sofia Bergh, Chrisovalantou Huridou, Rachel Y. Cheong, Barbara Baldo, Paul Günther Scheunemann, Marie‐Louisa Schoebel, Linda Holmquist Mengelbier, Elisabet Englund, Catriona McLean, Carsten Saft, Deniz Kirik, Maria Björkqvist, Glenda Halliday, Elisabeth Petrasch‐Parwez, Huu Phuc Nguyen, Jonasz Jeremiasz Weber, Åsa Petersén

**Affiliations:** ^1^ Translational Neuroendocrine Research Unit, Department of Experimental Medical Science Lund University Lund Sweden; ^2^ Department of Human Genetics, Medical Faculty Ruhr University Bochum Bochum Germany; ^3^ Institute of Medical Genetics and Applied Genomics, University of Tübingen Tübingen Germany; ^4^ Department of Neuroanatomy and Molecular Brain Research, Medical Faculty Ruhr University Bochum Bochum Germany; ^5^ Division of Pathology, Department of Clinical Sciences Lund University Lund Sweden; ^6^ Department of Pathology, Alfred Hospital Melbourne Victoria Australia; ^7^ Department of Neurology Huntington Centre NRW, St. Josef‐Hospital, Ruhr‐University Bochum Bochum Germany; ^8^ Huntington Center NRW, Ruhr‐University Bochum Bochum Germany; ^9^ Brain Repair and Imaging in Neural Systems (BRAINS), Department of Experimental Medical Science, Lund University Lund Sweden; ^10^ Brain Disease Biomarker Unit, Department of Experimental Medical Science Lund University Lund Sweden; ^11^ The Brain and Mind Centre and Faculty of Medicine and Health, School of Medical Sciences University of Sydney Sydney Australia; ^12^ Department of Psychiatry Skåne University Hospital Lund Sweden

**Keywords:** aggregation, huntingtin, huntingtin lowering, inclusions, neuropathology, SIDT2

## Abstract

The pathogenic mechanisms leading to neurodegeneration in Huntington disease (HD) are not fully understood but involve accumulation of toxic mRNA and protein products in the brain. Recent studies described an unconventional autophagic pathway involving DNA and RNA degradation through DNautophagy and RNautophagy that is regulated by the lysosomal protein SID1 transmembrane family member 2 (SIDT2). Interestingly, SIDT2 has been shown to bind to the expanded CAG repeat in the mutant huntingtin (mHTT) transcript and lower mHTT in vitro. The aim of the present study was to determine whether SIDT2 levels are altered in HD and whether manipulation of SIDT2‐mediated RNautophagy can alter HD pathology. We demonstrate a significant reduction of SIDT2 protein levels in the striatum and in the lateral hypothalamic area in postmortem HD brains compared to control cases without effects on SIDT2 mRNA levels. In frontal cortical postmortem HD tissue, we show a CAG‐repeat‐length‐dependent increase in the frequency of SIDT2‐immunoreactive intranuclear inclusions. In postmortem tissue of an HD case with Vonsattel grade 0, we demonstrate SIDT2‐ and mHTT‐immunoreactive inclusions not only in the frontal cortex, but also in the striatum and the lateral hypothalamic area. In the R6/2 mouse model of HD, we show that SIDT2 inclusions form at later stages than mHTT inclusions. Overexpression of SIDT2 using adeno‐associated viral vectors injected into the hypothalamus of R6/2 mice led to a reduction of mHTT inclusions in the lateral hypothalamic area. Similarly, in a neuronal cell model, overexpression of SIDT2 reduced soluble and insoluble mHTT exon 1 protein levels. Taken together, our results reveal novel pathology in clinical HD cases and in experimental models, characterized by the accumulation of SIDT2‐immunoreactive inclusions, while demonstrating the efficacy of overexpressing SIDT2 for lowering detrimental mHTT species. Targeting SIDT2‐mediated RNautophagy may offer a potential strategy to ameliorate the molecular pathology in HD.

## Graphical abstract

SIDT2‐immunoreactive inclusions are observed in the striatum, cerebral cortex, and hypothalamus in HD cases with different Vonsattel grades, and the frequency of SIDT2‐immunoreactive inclusions is associated with longer CAG repeats in the huntingtin gene. In HD mouse models (the R6/2 and AAV‐HTT‐79Q), SIDT2‐immunoreactive inclusions form after the formation of mutant huntingtin (mHTT) inclusions. Furthermore, overexpression of SIDT2 in HD mouse models and cell models provides support for beneficial effects on the clearance of mHTT.

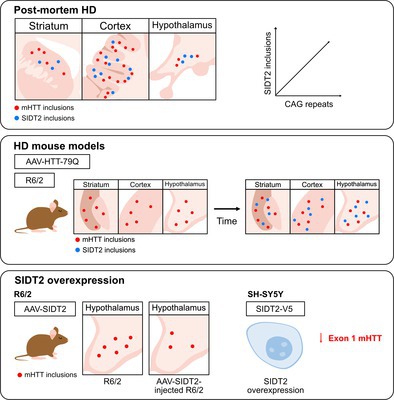

**Abbreviations**
AAV: adeno‐associated virus
ACC: anterior cingulate cortex
ASO: anti‐sense oligonucleotides
ARM: arginine‐rich motif
BSA: bovine serum albumin
DAB: 3,3′‐diaminobenzidine
DAPI: 4′,6‐diamidino‐2‐phenylindole
DPX: distyrene‐plasticizer‐xylene
DPBS: dulbecco's phosphate‐buffered saline
DTT: dithiothreitol
GAPDH: glyceraldehyde 3‐phosphate dehydrogenase
EDTA: ethylenediaminetetraacetic acid
HD: Huntington disease
HTT: huntingtin
HTTEx1: huntingtin exon 1
K48‐pUb: lysine‐48‐linked polyubiquitin chains
KPBS‐T: potassium phosphate‐buffered saline containing Triton X‐100
LC3B: MAP1LC3B (Microtubule‐associated proteins 1A/1B light chain 3B)
LDS: lithium dodecyl sulfate
mHTT: mutant huntingtin
p62: p62/SQSTM1 (sequestosome 1)
PAGE: polyacrylamide gel electrophoresis
PFA: paraformaldehyde
PBS: phosphate‐buffered saline
PVDF: polyvinylidene difluoride
qRT‐PCR: quantitative real‐time polymerase chain reaction
RT: room temperature
SID1: systemic RNA interference deficient‐1
SD: standard deviation
SDS: sodium dodecyl sulfate
S.E.M.: standard error of the mean
SIDT2: SID1 transmembrane family member 2
TBS‐T: Tris‐buffered saline containing Tween 20
UPS: ubiquitin‐proteasome system
wks: weeks

## 1 INTRODUCTION

Huntington disease (HD) is a fatal neurodegenerative disorder without any disease modifying therapy [1]. It is caused by an expanded CAG repeat in the huntingtin gene and there is a negative correlation between the number of CAG repeats and age of onset [1]. The huntingtin (HTT) protein is expressed in multiple brain areas, yet pathology evoked by mutant HTT (mHTT) is most pronounced in the striatum, the cerebral cortex, and in the hypothalamus, including the lateral hypothalamic area [2, 3, 4, 5]. The extent of striatal atrophy is classified according to the Vonsattel grading scale 0‐IV [6]. Neuropathological hallmarks include formation of mHTT‐containing neuronal inclusions in many HD affected brain regions [7] and in several animal models expressing different variants of mHTT [8]. The pathogenic mechanisms in HD are not fully understood but are likely to involve accumulation of toxic RNA and protein products due to alterations in protein degradation by the ubiquitin‐proteasome system (UPS) and autophagy [9, 10, 11, 12, 13].

Recent study has described an unconventional autophagic pathway involving DNA and RNA degradation, DNautophagy and RNautophagy is regulated by the lysosomal protein SID1 transmembrane family member 2 (SIDT2) [14, 15, 16, 17]. SIDT2 is the lysosomal vertebrate ortholog of the *Caenorhabditis elegans* RNA channel systemic RNA interference deficient‐1 (SID1) protein and is expressed in many cell types [18, 19]. SIDT2 can bind directly to DNA and RNA via an arginine‐rich motif (ARM) and mediates uptake into the lysosome for degradation. Interestingly, SIDT2 has been found to bind to exon 1 of the *HTT* transcript through its ARM in a CAG‐dependent manner [20]. Furthermore, overexpression of murine SIDT2 resulted in the degradation of *HTT* mRNA and reduced the levels of soluble and insoluble mHTT aggregates as assessed using filter trap and Western blot analyses in mHTT exon 1‐transfected Neuro2a cells [20]. The degradation of mHTT exon 1 is suggested to occur via SIDT2‐mediated RNautophagy. These results suggest that this novel pathway may play a role in HD and could be explored as a potential therapeutic target. Effects on SIDT2 or SIDT2‐mediated RNautophagy have not been studied previously in vivo in HD. In this study, we investigated whether the SIDT2 system is affected in HD by examining human postmortem tissue, multiple HD mouse models, and neuroblastoma cell cultures expressing exon 1 of mHTT. We show for the first time that SIDT2‐immunoreactive inclusions are formed in affected HD brain tissues, while SIDT2 protein levels are diminished in selected human brain areas with higher Vonsattel grades. Moreover, the occurrence of SIDT2‐positive inclusions was reproduced in two HD mouse models. Our results from SIDT2 overexpression both in vivo and in vitro further support beneficial effects on mHTT protein levels and inclusion load, paving the way for further developments targeting this pathway in HD.

## 2 MATERIALS AND METHODS

### 2.1 Overview of study design

In the present study, we examined the involvement of the SIDT2 system in human postmortem HD brains, in three HD animal models and in one in vitro system for HD. Frozen human brain tissue from 17 HD and 10 control cases were analyzed by Western blot and quantitative real‐time polymerase chain reactions (qRT‐PCR) in order to measure SIDT2 protein and mRNA levels (Tables 1 and 2). We assessed SIDT2 and mHTT inclusion formation in fixed postmortem human brain tissue from four HD patients with Vonsattel grades I‐IV and CAG repeats ranging from 42 to 66, one HD case with Vonsattel grade 0 with 44 CAG repeats and two control cases (Table 3). We investigated effects on SIDT2 inclusion formation in three animal models of HD: the R6/2 mouse, the BACHD mouse as well as in mice overexpressing fragments of mHTT or wild‐type HTT after hypothalamic injections of adeno‐associated viral (AAV) vectors. We also assessed effects of SIDT2 overexpression using AAV vectors injected into the hypothalamus of R6/2 mice. Finally, we analyzed effects of SIDT2 overexpression in an SH‐SY5Y neuroblastoma cell model transfected with mHTT exon 1 constructs, focusing on soluble protein levels, mHTT aggregate load, dysregulation of proteolytic pathways (specifically UPS and autophagy), and microscopy analysis for assessing subcellular protein distribution.

**TABLE 1:** Demographic data for HD and control cases used for cortical and striatal Western blot and qRT‐PCR analyses.

Case	Age/sex	CAG	DD	Cause of death	PMD	Brain	Grade	RIN: CTX	RIN: STR
*HD*									
HD1	68/m	44	13	Pneumonia	10	1184	3	7.5	6.8
HD2	69/f	42	20	Cardiorespiratory failure	2	1149	2	7.0	4.8
HD3	57/f	44	22	Pneumonia	22	800	4	4.8	5.9
HD4	61/m	43	17	HD endstage	17	1280	4	6.1	5.5
HD5	71/m	42	12	Myocardial infarct	41	1270	2	7.0	3.6
HD6	39/m	54	13	Cardiorespiratory failure	10	1047	4	7.4	5.7
HD7	39/f	46	11	HD endstage	36	680	3	8.0	7.9
HD8	58/m	46	11	HD endstage	32	1260	3	7.9	6.4
HD9	61/m	45	14	Sepsis	40	1500	4	7.6	3.6
HD10	62/m	43	12	HD endstage	22	1185	3	7.8	3.1
HD11	62/m	43	10	Pneumonia	24	1380	3	5.8	4.5
HD12	67/m	43	15	HD endstage	37	1200	2	6.9	4.6
HD13	67/m	45	15	Pneumonia	19	952	n.d.	6.6	3.0
HD14	71/m	40	10	Pneumonia	39	1150	3	4.2	3.2
HD15	72/m	43	33	Pneumonia	22	940	2	5.0	3.4
HD16	74/m	40	12	Cardiorespiratory failure	27	1475	2	6.5	6.0
HD17	77/m	41	20	Pneumonia	9	1018	4	7.6	7.0
Mean ± SD	63 ± 11 m: 14 f: 3		15 ± 6		24 ± 12	1114 ± 219		6.7 ± 1.2	5.0 ± 1.5
*Control cases*									
C1	69/m			Pulmonary embolism	24	1290		6.9	6.7
C2	67/f			Pulmonary embolism	26	1298		7.5	6.1
C3	57/m			Ischemic heart disease	48	1532		6.6	7.2
C4	73/m			Ischemic heart disease	43	n.d.		6.4	6.8
C5	64/m			Ischemic heart disease	32	1335		6.8	2.5
C6	64/m			Ischemic heart disease	24	1492		7.5	7.7
C7	66/f			Metastatic carcinoma	43	1233		6.8	6.5
C8	69/m			Ischemic heart disease	34	1240		6.6	6.5
C9	76/m			Aortic aneurysm	46	1459		7.8	7.6
C10	78/m			Ischemic heart disease	46	1471		6.9	6.5
Mean ± SD	68 ± 6 m: 8 f: 2				37 ± 10	1372 ± 116		7.0 ± 0.5	6.4 ± 1.5

*Note*: Age is indicated in years. PMD (postmortem delay) is indicated in h. DD (disease duration) is indicated in years. Brain (total brain weight) is indicated in g. Grade refers to Vonsattel grade for neuropathological classification of HD [53].

Abbreviations: CTX, cerebral cortex; n.d. not determined; RIN, RNA integrity number; STR, striatum.

**TABLE 2:** Demographic data for HD and control cases used for Western blot analyses of the lateral hypothalamic area.

Case	Age/sex	CAG	DD	Cause of death	PMD	Brain	Grade
HD							
HD1	68/m	44	13	Pneumonia	10	1184	3
HD2	69/f	42	20	Cardiorespiratory failure	2	1149	2
HD3	57/f	44	22	Pneumonia	22	800	4
HD4	61/m	43	17	HD endstage	17	1280	4
Mean ± SD	64 ± 6 m: 2 f: 2		18 ± 4		13 ± 9	1103 ± 210	
Control cases							
C1	69/m			Pulmonary embolism	24	1290	
C2	67/f			Pulmonary embolism	26	1298	
C3	57/m			Ischemic heart disease	48	1532	
Mean ± SD	64 ± 4 m: 2 f: 1				32 ± 13	1373 ± 138	

*Note*: Age is indicated in years. PMD (postmortem delay) is indicated in h. DD (disease duration) is indicated in years. Brain (total brain weight) is indicated in grams. Grade refers to Vonsattel grade for neuropathological classification of HD [53].

Abbreviation: HYP, hypothalamus.

**TABLE 3:** Demographic data for HD and control cases used for immunohistochemical SIDT2 and HTT analyses.

Case	Age/sex	CAG	DD	Cause of death	PMD	Brain	Grade
HD1	67/m	42/17	7	Cardiac failure	74	1343	1–2
HD2	55/f	44/17	12	Suicide	110	1284	2–3
HD3	40/f	50/18	15	Pneumonia	20	1070	3
HD4	32/f	66/20	14	Pneumonia	44	695	4
HD5	52/m	43/19	0	Cardiac arrest	96	1282	0
C1	69/f	21/15	n/a	Cardiac failure	12	1282	n/a
C2	79/m	25/20	n/a	Cardiac failure	27	1364	n/a

*Note:* Age is indicated in years. DD (disease duration) is indicated in years. PMD (postmortem delay) is indicated in h. Brain (total brain weight) is indicated in g. Grade refers to Vonsattel grade for neuropathological classification of HD [53].

### 2.2 Methods related to postmortem human brain tissue

#### 2.2.1 Human postmortem brain tissue

Tissue from the caudate nucleus of the striatum and frontal cortex from 17 HD and 10 control cases were collected in blocks and frozen on dry ice upon autopsy and stored at −80°C for Western blot analyses and qRT‐PCR analyses as reported previously [21]. Tissues from the entire hypothalamus from four HD and three control cases were frozen on dry ice upon autopsy and stored at −80°C. The lateral hypothalamic area was dissected as previously described [21] and used for Western blot analysis in the present study. Demographic data are shown in Tables 1 and 2. This set of human postmortem tissue was obtained from Victorian and Sydney Brain Banks in Australia, after approval of the project by their Scientific Review Committee (PID167). All persons had given their informed consent prior to the donation of their brains, and the brain donor programs were approved by Institutional Human Research Ethics Committees.

Paraformaldehyde (PFA)‐fixed anterior cingulate cortex (ACC) from four HD and two control cases were used for immunohistochemistry. The two control patients had no clinical history of neurological or psychiatric diseases and the HD mutation was genetically excluded. Demographic data is shown in Table 3. This set of human postmortem tissue was obtained from the Huntington brain collection of Bochum (EPP and CS). All individuals had given written consent to examine the brains for research purposes. The study was approved by the Ethic Committee of the Medical Faculty of the Ruhr‐University Bochum, Germany (Reg. No. 17‐5939).

Formalin‐fixed tissue from the frontal cortex, the striatum, and the hypothalamus of an HD case with Vonsattel grade 0 was obtained from the Department of Neuropathology in Lund, Sweden [22]. Demographic data is shown in Table 3. The analysis was approved by the regional ethical review board at Lund University (reference number 2014/466), and written informed consent was obtained from the patient. All analyses performed on human postmortem tissue in Sweden were approved by the Swedish Ethical Review Authority (reference number 2022–05348‐01).

#### 2.2.2 Western blots

Human postmortem tissue from frontal cortex, the caudate nucleus of the striatum, and the lateral hypothalamic area were lysed in 1:10 weight/volume in lysis buffer (50 mM NaCl, 100 mM Tris–HCl pH 7.4, 1 mM EDTA, 1% SDS) supplemented with protease and phosphatase inhibitors (Roche). The homogenization of the tissue was performed by sonication (15× for 1 s at 40 Hz). The samples were then incubated for 15 min on ice and consecutively spun at 13,000 rpm for 10 min at 4°C. Total protein concentration was determined using the DC Protein Assay Kit (Bio‐Rad) according to manufacturer's instructions. Before loading on the SDS‐PAGE, the samples were boiled at 95°C for 5 min in the presence of Laemmli Loading Buffer (Bio‐Rad). 40 μg of total protein was loaded on each well on a 4–15% gradient polyacrylamide gel (Bio‐Rad Mini‐PROTEAN TGX Precast Gels). The samples were run for 30 min at 90 V, followed by 1 h at 120 V. Proteins were then transferred onto the PVDF membrane (Bio‐Rad) using the Trans‐Blot Turbo Transfer System (Bio‐Rad) for 7 min at 25 V. Blocking was performed for 1 h at room temperature (RT) using 5% skimmed milk in Tris‐buffered saline containing 0.1% Tween 20 (Sigma) (TBS‐T), followed by incubation overnight at 4°C with a primary antibody against SIDT2 (at 1:1000, made in rabbit, Abcam ab85847 for cortical and striatal samples; at 1:1000, made in rabbit, Abnova PAB27211 for hypothalamic and striatal samples, due to discontinuation of the Abcam antibody) or β‐actin (at 1:10000, made in mouse) diluted in 2% milk in TBS‐T. After three 10 min washes in TBS‐T, the membranes were incubated for 1 h at RT with a secondary antibody (mouse anti‐rabbit, Santa Cruz Biotechnology, sc‐2357 at 1:100; or donkey anti‐mouse, ab6789 at 1:10000). After three 10 min washes in TBS‐T, the membranes were developed using enhanced chemiluminescence (BioRad). The images were acquired with the VersaDoc instrument (Bio‐Rad). Band intensities were quantified using the ImageLab software (Bio‐Rad) and expressed as a ratio relative to β‐actin.

#### 2.2.3 qRT‐PCR

Total RNA was isolated from the tissue samples using RNeasy Lipid Tissue Kit (Qiagen) with an on‐column DNase digestion (RNase‐free DNase set, Qiagen) according to supplier's recommendations. RNA quantity was measured on a NanoDrop 2000 spectrophotometer (Thermo Scientific). RNA integrity number, an indicator for appropriate preservation of RNA integrity, was used to assess the RNA quality of the human postmortem tissue [23]. RNA samples were analyzed by SCIBLU Genomics, Affymetrix unit at Lund University using Agilent 2100 Bioanalyze and RNA integrity was determined for all samples before proceeding with the analyses as reported previously [21] (Table 1). cDNA was generated using random hexamer primers and SuperScript IV Reverse Transcriptase (Invitrogen) according to supplier's recommendations. qRT‐PCR reaction was performed on a LightCycler 481 in a two‐step protocol using SYBR Green I Master mix (Roche). The specificity of the amplification was determined by melting curve analysis. Data were quantified using the ΔΔCT‐method and were normalized to the expression of the two housekeeping genes β‐actin (*ACTB*) and glyceraldehyde 3‐phosphate dehydrogenase (*GAPDH*). All primers were designed with Beacon DesignerTM (Premier Biosite). All values are presented as ratios to the mean of the control group. The primer sequences used for the gene expressions analyses were (5′‐3′): *ACTB* (Fwd: ATATGAGATGCGTTGTTA, Rev.: AAGTATTAAGGCGAAGAT), *GAPDH* (Fwd: CTCTGGTAAAGTGGATATTGT, Rev.: GGTGGAATCATATTGGAACA), and *SIDT2* (Fwd: TGAACGTCCTGAACAAGCAG, Rev.: CACAGGGTTCGTTCCACTTT).

#### 2.2.4 Immunohistochemistry of human postmortem brain tissue

We examined three frontal 80 μm vibratome sections of the ACC per HD and control brains (Table 3, HD1‐4; C1 and 2), all of which were located at the same supra‐callosal level. All human brains were fixed by immersion in 4% PFA. For immunohistochemistry all sections were rinsed free floating in phosphate buffered saline (PBS) and subjected to SIDT2 and EM48 antibodies according to a protocol described previously [24]. Briefly, the sections were treated with 1% NaBH_4_ and incubated for 30 min in PBS with 10% normal goat or horse serum and 0.3% Triton X‐100 (Serva 37,240), followed by an incubation with antibodies against SIDT2 (1:1000, made in rabbit, Abnova PAB272) or HTT (1:1000, anti‐mouse, EM48, MAB5374, Merck Millipore) for 72 h at 4°C in the same solution. After rinsing in PBS and a preincubation with 0.1% bovine serum albumin in PBS for 1 h the sections were incubated overnight at 4°C with a biotinylated goat anti‐rabbit secondary antibody (CA 94010, Vector Laboratories) or horse anti‐mouse antibody (Thermo Fisher Scientific). After blocking in PBS‐albumin at RT, sections were incubated with the avidin‐biotinylated peroxidase complex (Vector Laboratories) for another 3 h at RT. Peroxidase activity was visualized with 3,3′‐diaminobenzidine (DAB). The reaction was controlled microscopically, with a maximum of 15 min. Finally, sections were mounted on Superfrost Plus slides (Thermo Fisher Scientific), air‐dried for 1.5 h, dehydrated and coverslipped. As negative controls for HD‐specific immunolabeling, we used cases without known history of neurological or psychiatric diseases. HD was excluded by genetic testing of postmortem blood in these controls. In order to exclude the possibility of nonspecific staining, we performed omission of primary antibodies in normal goat or horse serum, which both lead to absence of immunoreactivity in the sections.

For immune electron microscopic analyses, sections were treated as described above with the omission of Triton X‐100. These sections were photodocumented after the immunohistochemical procedure for later orientation, postfixed with 1% osmium tetroxide in PBS for 1 h, dehydrated and flat‐embedded in Araldite (Serva). Alternating semi‐ and ultrathin sections were cut with a Leica Ultracut R microtome. Semithin sections (0.75 μm) were lightly counterstained with 1% toluidine blue 0 (T3260, Sigma‐Aldrich), ultrathin sections (100 nm) contrasted with 5% aqueous uranyl acetate (E22400, Science Services) for 10 min and 2% aqueous lead citrate (228,621, Sigma‐Aldrich) for 5 min. Ultrathin sections were viewed by the LVEM25 (Delong Instruments, Brno, Czech Republic).

For the HD case with Vonsattel grade 0, formalin‐fixed coronal tissue blocks of the striatum, the cerebral cortex and the hypothalamus were cryoprotected in 30% sucrose and serially cut at a thickness of 50 μm on a freezing microtome in 15 series. Briefly, antigen retrieval was performed on free‐floating sections with Tris‐EDTA buffer with pH 9.0 at 80°C for 1 h, quenched in 3% hydrogen peroxide and 10% methanol for 30 min and blocked in TBS‐T containing 5% serum (from the same species as the corresponding secondary antibody was raised in) for 1 h at RT. Subsequently, the sections were incubated overnight using primary antibodies against SIDT2 (1:2500, anti‐rabbit, PAB27211, Abnova) and HTT (1:200, anti‐mouse, EM48) in antibody solution containing 1% BSA and TBS‐T. The following day, the sections were incubated with secondary antibody for 1 h at RT (1:200 in 1% BSA TBS‐T). The sections were visualized using hydrogen peroxide and DAB development. Finally, the sections were mounted on chromatin‐gelatin coated glass slides and coverslipped with Distyrene‐Plasticizer‐Xylene (DPX) (Sigma Aldrich) after a series of dehydration steps in ascending concentrations of ethanol and xylene.

For immunofluorescence microscopy, dissected ACC were rinsed in PBS, immersed overnight in 30% saccharose in PBS, then shock‐frozen and immunostained as previously described [25]. Briefly, immunohistochemistry was performed on cryosections (14 μm) mounted on Superfrost Plus slides and dried for 1.5 h at 40°C. Sections were simultaneously incubated with antibodies against SIDT2 (1:200 anti‐rabbit, Abnova PAB27211) and HTT (1:300 anti‐mouse, EM48) overnight at 4°C and then treated with secondary antibodies conjugated with Alexa Fluor 594 or Alexa Fluor 488 (1:500) for 2 h at RT. To reduce unspecific background staining, we applied True Black autofluorescence quencher (1:20 in 70% ethanol) (BIOZOL Diagnostica Vertrieb, Germany) for 1 min. Finally, all sections were counterstained with the fluorescent dye 4',6‐diamidino‐2‐phenylindole (DAPI; 1:1000; Roche Diagnostics). All sections were documented in a confocal fluorescence microscope (Nikon Spinning Disk) using a 60× water magnification immersion objective. For immunofluorescent staining of the HD case with Vonsattel grade 0, antigen retrieval was performed in Tris‐EDTA buffer with pH 9.0 at 80°C for 1 h; samples were blocked in potassium phosphate‐buffered saline in 0.25% Triton X‐100 (KPBS‐T) containing 5% donkey serum for 1 h. Subsequently, the sections were incubated overnight using primary antibodies against SIDT2 (1:300, anti‐rabbit, PAB27211, Abnova) and HTT (1:100 anti‐mouse, EM48) in KPBS‐T containing 5% donkey serum. Sections were labelled with biotin‐conjugated secondary antibody (1:200, donkey anti‐rabbit) followed by Alexa Fluor 647‐conjugated streptavidin and Alexa Fluor 488‐conjugated secondary antibody (1:200, donkey anti‐mouse, Jackson Laboratories). Sections were mounted on chromatin‐gelatin‐coated glass slides and coverslipped using VECTASHIELD® Mounting Medium (Vector Laboratories).

#### 2.2.5 Image acquisition

Photodocumentation of vibratome and semithin sections was performed using an Olympus Microscope BH‐2 equipped with an Olympus DP28‐CU Camera (Olympus Optical) and the computer‐assisted cellSense Entry program version 3 (Olympus). Images of 80 μm vibratome sections were documented on Z‐stacks created with Zerene Stacker (Zerene Systems, Cherrywood, Loop Richland, WA, USA) in ImageJ to show the representative distribution of SIDT2‐immunoreactive inclusions and HTT (mEM48)‐immunoreactive inclusions. Images used in the same figure were adapted for brightness and contrast. Brightfield and fluorescence image acquisitions of the HD case with Vonsattel grade 0 were performed using a Zeiss Axio Imager M2 microscope (Zeiss, Göttingen, Germany) or a Zeiss Axiocam 305 color camera. For each figure, images were acquired with consistent settings, and contrast was optimized by adjusting the histogram using the Zeiss Zen 3.7 software. Image panels were created and edited using Affinity Designer 1.10 (Affinity).

### 2.3 Methods related to animal models

#### 2.3.1 Animal models

All mice were housed in cages of 2–5 animals and maintained in a 12‐h light/dark cycle with ad libitum access to food and water. All described experimental procedures performed in animals were approved by the Regional Ethical Committee in Malmö/Lund, Sweden under permit number 17113/2022. Brain tissue from the R6/2 mouse, a well‐established transgenic model of HD expressing exon 1 of the mutant *HTT* gene [26] was used for neuropathological analyses at 6 (*n* = 4/genotype) and 16 weeks of age (*n* = 6 R6/2 and *n* = 2 wild‐type littermates). The R6/2 mice used in this study had a CAG repeat size range between 276 and 296 resulting in a slower disease progression compared to R6/2 mice with 150 CAG repeats [27, 28]. Brain tissue from the BACHD mouse, another well‐established transgenic HD mouse expressing the full‐length mHTT with around 97 glutamines [29] in the colony at Lund University [30], was used for neuropathological analyses at 6 months of age (*n* = 6 for BACHD and *n* = 3 for wild‐type littermates). Brain tissue from mice overexpressing fragments of 853 amino acids of HTT with either 79Q (mHTT) or 18Q (wild‐type HTT) after hypothalamic injections of AAV vectors at 6 weeks of age in the experiments described in Hult et al., 2011 [31] was used for neuropathological analyses at 6 and 18 weeks post‐injection (*n* = 1/group).

In order to test the effects of overexpression of SIDT2 in the R6/2 mice, an AAV vector was produced to overexpress human SIDT2 (AAV‐SIDT2). The transgene sequence was regulated under the synapsin 1 promoter and flanked with simian virus 40 polyadenylation signal sequence between the inverted terminal repeat from AAV2 genome and packaged in a AAV5 capsid, as previously described [31, 32]. Stereotactic injections of 0.5 μL of the AAV vectors at 1.0 E14 genome copies/ml was performed bilaterally in the hypothalamus as previously described [33] at the following coordinates: anterior/posterior = −0.7 mm, medial/lateral: ±0.55 mm, ventral/dorsal = −5.2 mm in four R6/2 mice at 6 weeks of age. The stereotactic coordinates were calculated relative to the bregma and dura. Neuropathological analyses were performed at 10 weeks post‐injection, that is, at 16 weeks of age and AAV‐SIDT2 injected R6/2 mice were compared to uninjected R6/2 mice of the same age (*n* = 4–5/group).

#### 2.3.2 Immunohistochemistry of mouse brain tissue

Mice were sacrificed using sodium pentobarbital (~150 mg/kg i.p.) and transcardially perfused first with saline and then pre‐cooled 4% PFA. Brains were removed, and then post‐fixed for 24 h in 4% PFA and placed in 25% sucrose for cryoprotection. Brains were sectioned frozen in the coronal plane at a thickness of 30 μm in six series. The sections were processed as previously described [33]. Primary antibodies used in three separate series of sections were against SIDT2 (1:2000, anti‐rabbit, PAB27211, Abnova), the N‐terminus of HTT (1:500, anti‐goat, sc‐8767, Santa Cruz Biotechnology; or 1:100 mEM48) or hypocretin (1:4000, anti‐rabbit, H‐003‐30, Phoenix Pharmaceuticals). Sections were labelled with suitable secondary antibody (1:200, anti‐rabbit, anti‐goat or anti‐mouse) and visualised with DAB using hydrogen peroxidase. Lastly, sections were mounted on chromatin‐gelatin coated glass slides, dehydrated in an ethanol and xylene series and coverslipped with DPX (Sigma Aldrich).

#### 2.3.3 Estimation of the number of hypocretin‐immunopositive neurons and mHTT inclusions

Images were captured at consistent magnification and brightness settings on a Zeiss PrimoStar microscope (Zeiss) and quantified in ImageJ 1.53t. Images of EM48‐immunoreactive mHTT inclusions were acquired bilaterally in the superior lateral part of the fornix at bregma −1.70 mm using a 40×/0.6 NA objective. Images were made binary and thresholded (0–130). Watershed segmentation was applied to separate overlapping inclusions. Inclusions with a pixel size range of 6–250 and a circularity value between 0.20 and 1.00 were automatically counted. The total number of mHTT inclusions was then calculated per square mm. Images of hypocretin‐immunoreactive neurons were acquired bilaterally in the lateral hypothalamic area at a bregma range of −1.06 to −2.30 mm using a 4×/0.1 NA objective. Images were made binary and thresholded (0–48). Watershed segmentation was applied, and neurons with a pixel size range of 15–infinity and a circularity value 0.10–1.00 were counted. The total number of neurons was estimated by multiplying the total cell count by the number of series. Image acquisition was performed as previously described for the HD case with grade 0.

### 2.4 Methods related to cell culture experiments

#### 2.4.1 Cell culture expression constructs

To overexpress human SIDT2, pcDNA3.1 vectors carrying C‐terminally V5‐tagged SIDT2 (UniProt ID: Q8NBJ9‐1) were synthesized (BioCat, Heidelberg, Germany). A pcDNA3.1 plasmid without an insert served as an empty vector control. The HTT exon 1‐coding pCMV HTT_Ex1_ 16Q and pCMV HTT_Ex1_ 72Q constructs were a gift from Hilal A. Lashuel (EPFL, Lausanne, Switzerland).

#### 2.4.2 Cell cultures

All cell culture experiments were conducted in SH‐SY5Y (ATCC: CRL‐2266) cells, maintained in Dulbecco’s modified Eagle medium (DMEM) GlutaMAX™ supplemented with 10% foetal bovine serum, 1% non‐essential amino acids and 1% Antibiotic‐Antimycotic (A/A) (all Gibco®, Thermo Fisher Scientific) in 5% CO_2_ at 37°C. Cells were transiently transfected for 72 h with Lipofectamine 3000 Transfection Reagent (Thermo Fisher Scientific) according to the manufacturer's protocol.

#### 2.4.3 Protein extraction from cells

For generating protein extracts, cells were dissociated by trypsinization and centrifuged for 5 min at 500×*g* in a pre‐cooled (4°C) centrifuge. Supernatant was discarded and cell pellets were washed with cold 1× Dulbecco's phosphate‐buffered saline (DPBS) for 2.5 min at 500×*g* and 4°C. Cell pellets were resuspended in RIPA buffer (50 mM Tris pH 7.5, 150 mM NaCl, 0.1% (w/v) SDS, 0.5% (w/v) sodium deoxycholate, 1% (v/v) Triton X‐100, and 10% (v/v) glycerol) supplemented with cOmplete™ protease inhibitor cocktail and PhosSTOP™ phosphatase inhibitor (Roche) and ultrasonicated using a Sonopuls ultrasonic homogenizer (Bandelin electronic) at 10% power and 10% pulse duration for 3 s. Protein concentrations were determined spectrophotometrically in a microtiter plate using Bio‐Rad Protein Assay Bradford reagent (Bio‐Rad Laboratories).

#### 2.4.4 Western blots of cell cultures samples

For protein extracts obtained from cells, Western blotting was performed according to standard procedures. In brief, 25 μg of total protein was mixed in a ratio 3:1 with 4× LDS sample buffer (1 M Tris pH 8.5, 43% (v/v) glycerol, 8% (w/v) LDS, 2 mM EDTA, 0.1% (v/v) Orange G) with 100 mM of dithiothreitol (DTT) and heat‐denatured at 70°C for 10 min. Protein samples were then electrophoretically separated on 10% Bis‐Tris gels using MOPS electrophoresis buffer (50 mM MOPS, 50 mM Tris pH 7.7, 0.1% (w/v) SDS, and 1 mM EDTA). Separated proteins were transferred onto Amersham™ Protran™ Premium 0.2 μm nitrocellulose membrane (Cytiva) using a Bicine/Bis‐Tris transfer buffer (25 mM Bicine, 25 mM Bis‐Tris pH 7.2, 1 mM EDTA, and 15% (v/v) methanol) at 80 V and a maximum of 250 mA for 2 h.

#### 2.4.5 Filter retardation assay

Detection of SDS‐insoluble proteins was performed as previously described [34]. Briefly, 3 μg of total protein was diluted in 1× DPBS containing 2% (w/v) SDS and 50 mM DTT and heat‐denatured for 5 min at 95°C. Using a Minifold® II Slot Blot system (Schleicher & Schuell), an Amersham™ Protran™ 0.45 μm nitrocellulose membrane (Cytiva) was equilibrated with 1× DBPS containing 0.1% (w/v) SDS, and samples were subsequently filtered through applying a vacuum. Afterwards, slots were washed once with 1× DBPS containing 0.1% (w/v) SDS and twice with 1× DBPS before the standard immunodetection protocol was applied.

#### 2.4.6 Visualization of Western blots and filter retardation assays of cell culture samples

Membranes obtained from Western blotting or filter retardation assays of cell culture samples were blocked with 5% (w/v) skim milk powder (Merck) in 1× TBS (10 mM Tris pH 7.5, 150 mM NaCl) at RT and subsequently probed with primary antibodies diluted in 1× TBS‐T (TBS with 0.1% Tween 20) at 4°C overnight. A detailed listing of used primary antibodies can be found in Table S1. Afterwards, membranes were washed with 1× TBS‐T and probed for 1 h at RT with the respective fluorescently labeled secondary IRDye® antibodies (goat anti‐mouse 680LT, goat anti‐mouse 800CW, and goat anti‐rabbit 800CW;926‐68020, 926‐32210, 926‐32211, all 1:5000; LI‐COR Biosciences). After incubation, membranes were washed a final time with 1× TBS‐T. Fluorescence signals were detected and quantified using the LI‐COR ODYSSEY® FC and the Image Studio 5.2 software (both LI‐COR Biosciences).

#### 2.4.7 Immunocytostaining and fluorescence microscopy

For immunocytochemistry, SH‐SY5Y cells were seeded on poly‐L‐lysine (P4707, Sigma‐Aldrich) pre‐coated 8‐well chamber slides (80,841, Ibidi), and transfected after 24 h. After 96 h, cells were fixed with 4% (w/v) PFA in 1× DPBS. For blocking and permeabilization, cells were incubated for 1 h at RT in 10% (w/v) bovine serum albumin, 0.5% (v/v) Triton X‐100, and 0.02% (w/v) NaN_3_ in 1× DBPS. Subsequently, cells were probed with primary antibodies anti‐V5 (made in mouse, 1:500; clone SV5‐Pk1, #R960‐25, Thermo Fisher Scientific) and anti‐HTT (made in rabbit, 1:500; clone EPR5526, ab109115, Abcam) diluted in antibody diluent (1% (w/v) bovine serum albumin, 0.5% (v/v) Triton X‐100, 0.02% (w/v) NaN_3_ in 1× DBPS) at 4°C overnight. The following day, cells were washed and incubated for 1 h at RT with goat anti‐mouse IgG AlexaFluor™ 594 or goat anti‐rabbit AlexaFluor™ 488 (both 1:500; A32742, A27034, Thermo Fisher Scientific). After washing, cells were mounted and counterstained with VECTASHIELD® Antifade Mounting Medium with DAPI (H‐1200, Vector Laboratories). Epi‐fluorescence images were taken at a 630× (Plan‐Neofluar 63×/1.4 oil objective) magnification on an Axioplan 2 imaging microscope equipped with an ApoTome and an AxioCam MRm camera, using the AxioVision 4.3 imaging software (all Zeiss, Oberkochen, Germany).

### 2.5 Statistical analyses

Data on human and mouse tissues was analyzed using independent samples Mann–Whitney *U* test or Students *t* test in SPSS Inc. 18.0. Statistical significance was set at *p*‐value <0.05. Graphs were generated using GraphPad Prism 10.2 (GraphPad Software). Data are presented as mean ± standard deviation (SD) or median with range. Statistical analyses of cell culture‐based experiments were performed using GraphPad Prism 10.1.1 (GraphPad Software). Results are presented as bar charts representing mean + standard error of mean (SEM). Two‐way ANOVA with the respective post‐hoc analyses were applied. Statistical significance was set at a *p*‐value ≤0.05. Further information is provided in the respective figure legends.

## 3 RESULTS

### 3.1 SIDT2 levels are reduced in striatum and lateral hypothalamus in human HD cases

**FIGURE 1:**
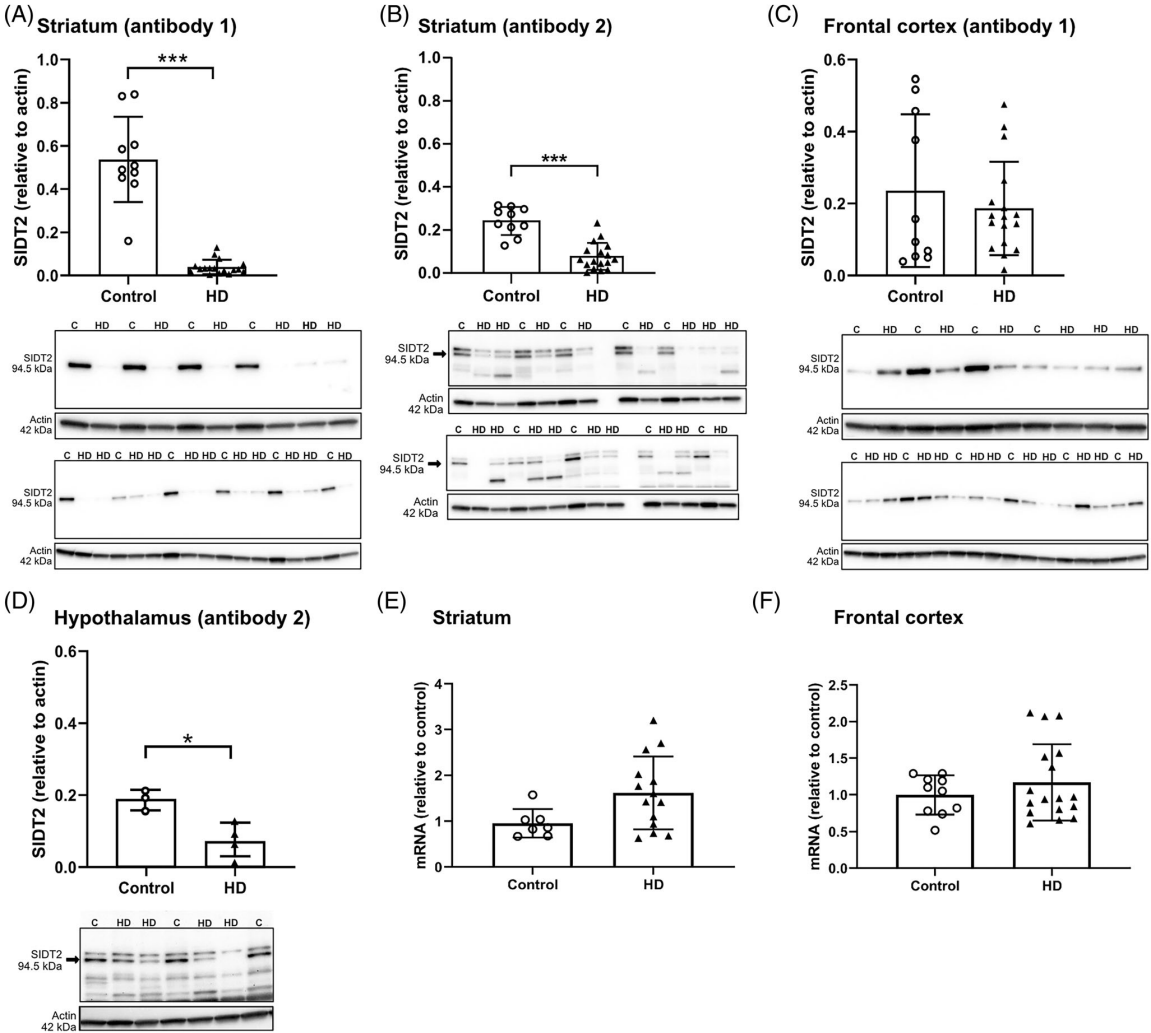
Reduced SIDT2 protein levels in the striatum and the lateral hypothalamic area in HD cases. Western blots of SIDT2 in the striatum (A, B), the cerebral cortex (C), and the lateral hypothalamic area (D) from HD cases compared to age‐ and sex‐matched control cases. Antibody 1 is Abcam ab85847, and antibody 2 is Abnova PAB27211 that replaced the first antibody in this study due to the discontinuation of antibody 1 at the company level. qRT‐PCR shows no differences in mRNA levels of SIDT2 in the striatum (E) and cerebral cortex (F) between HD and control cases. Data are expressed as mean ± SD. Student's *t*‐test, * = *p* < 0.05.

First, we investigated SIDT2 protein levels in human postmortem tissue from HD and control cases. Western blot analysis revealed a significant reduction of SIDT2 protein levels in the caudate nucleus of the striatum (93% with antibody 1 and 68% reduction with antibody 2) and a significant 67% reduction of SIDT2 in the lateral hypothalamic area of HD cases compared to control cases (Figure 1A,B,D and S1–S4). No differences in SIDT2 protein levels were detected between HD and control cases in the frontal cortex (Figure 1C). mRNA levels of SIDT2 in the striatum or the frontal cortex did not differ between HD and control cases (Figure 1E,F). This data indicate a depletion of soluble SIDT2 protein in the striatum and, to a lesser extent, in the lateral hypothalamic area during later stages of HD, independent of its transcription.

### 3.2 CAG‐dependent frequency of SIDT2‐immunoreactive inclusions in the HD ACC

**FIGURE 2:**
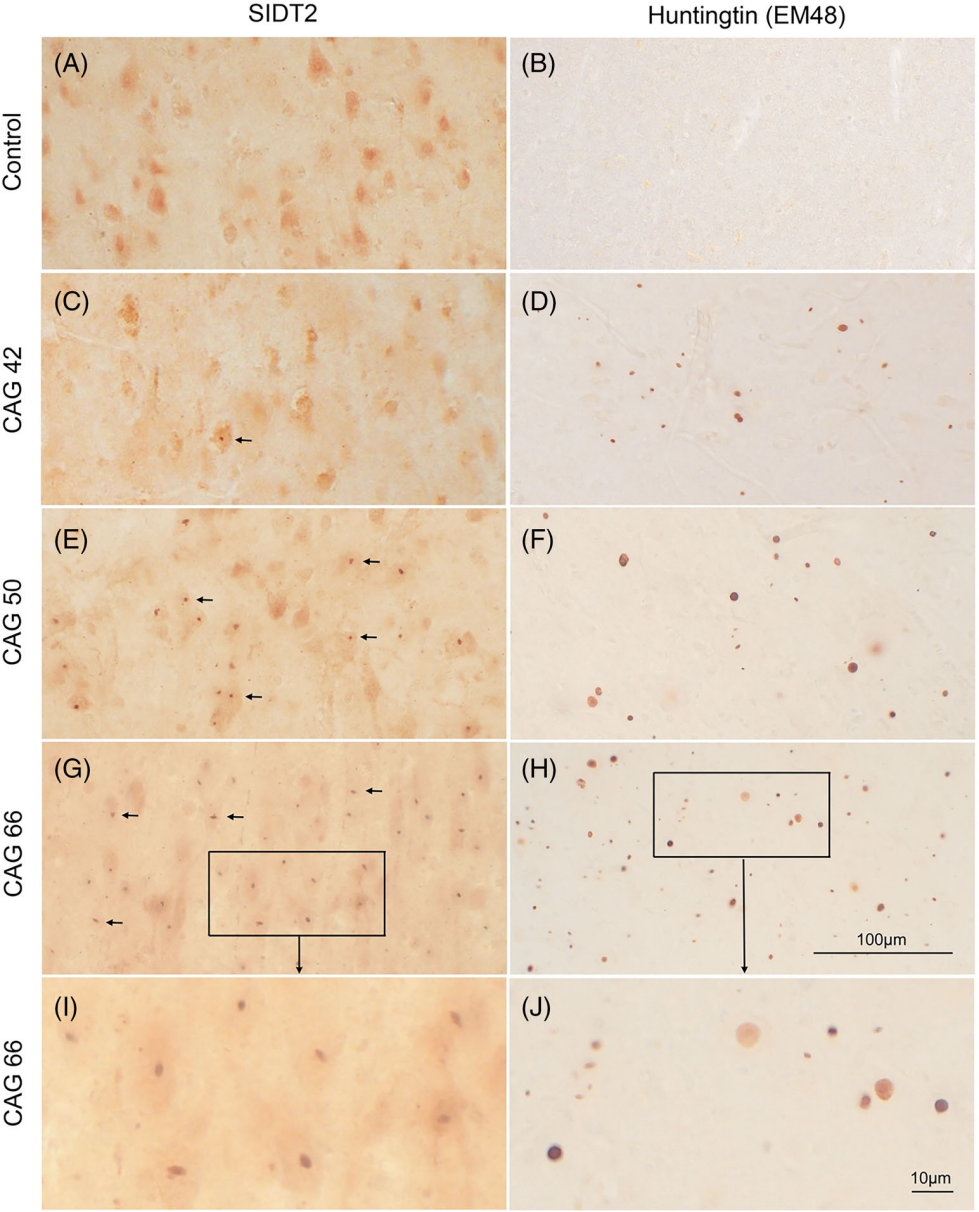
Formation of SIDT2‐immunoreactive CAG‐repeat dependent inclusions in human HD cortex. SIDT2 immunoreactivity is localized in multiple neuronal nuclei, cytoplasm, and dendrites of HD and in control anterior cingulate cortex (A, C, E, G, I). In the HD case with 42 CAG repeats, there are single SIDT2 intranuclear inclusions (C; black arrow) not observed in the control (A). HD cases with 50 and 66 CAG repeats present abundant inclusions (E, G, I). EM48‐immunoreactive mHTT inclusions, lacking in the control (B), also become denser in distribution with increasing CAG length (D, F, H), but in contrast to the SIDT2 inclusions, the EM48‐immunopositive mHTT inclusions are heterogeneous in size and form (J) and mainly localized in the cytoplasm and neuropil. Intranuclear EM48‐immunoreactive inclusions are rare.

Given the preserved SIDT2 protein levels in the HD cortex, and since the ACC is characterized by frequent mHTT inclusions unlike striatal areas that display greater neuronal loss in later stages [35], we examined SIDT2 immunoreactivity in the ACC of postmortem human HD cases. SIDT2 immunoreactivity was observed in numerous neurons in both HD and control cases with a similar distribution pattern (Figure 2A,C,E,G,I). The reactivity was detected in the nucleus, cytoplasm, and dendritic processes, varying in expression and intensity. Interestingly, HD cases showed SIDT2‐immunoreactive intranuclear inclusions of uniform size when compared to control cases. In HD patients with CAG repeat lengths of 42 and 45, SIDT2 inclusions were rare (Figure 2C, data for CAG 45 not shown), whereas in HD individuals with CAG repeats of 50 and 66, intranuclear inclusions were frequent, suggesting that the frequency of SIDT2 intranuclear inclusions is dependent on the CAG repeat length (Figure 2E,G,I). Most inclusions were localized in layers V and VI of the ACC.

**FIGURE 3:**
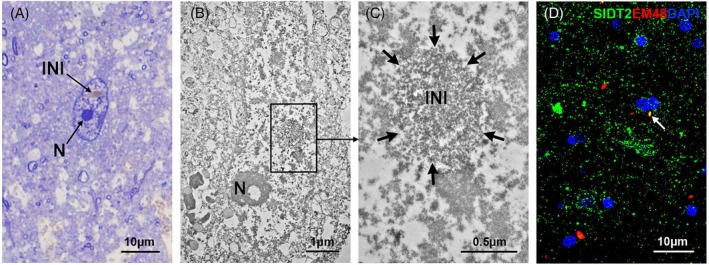
SIDT2 transmission electron and fluorescence microscopy in human HD cortex. Peroxidase immunohistochemistry (A–C) shows a large pyramidal cell in layer V of the anterior cingulate cortex with a SIDT2‐immunoreactive intranuclear inclusion (INI) clearly distinguished from the nucleolus (N) as illustrated in a semithin section counterstained with Toluidine‐blue (A) and a transmission electron microscopic picture (B). The enlargement (C) confirms membrane‐less INI indicated by black arrows. (D) Triple fluorescence staining with SIDT2 (green), EM48 (red), and DAPI (blue) exhibits only rare co‐localization of mHTT and SIDT2 (white arrow).

We then compared SIDT2 immunoreactivity with the distribution of mHTT inclusions in the ACC. The density of EM48‐immunopositive inclusions also increased with the CAG repeat length (Figure 2, D, F, H, J), confirming previous observations in cortical areas [36]. However, in contrast to SIDT2‐immunoreactive intranuclear inclusions, EM48‐immunoreactive inclusions were heterogeneous in size and form, mainly localized in the cytoplasm and neuropil, while intranuclear inclusion bodies were rare. To elucidate the subcellular composition of SIDT2 intranuclear inclusions, transmission immunoelectron microscopy was performed, showing dense and membrane‐less SIDT2‐immunoreactive intranuclear inclusions that were clearly distinguishable from the nucleolus (Figure 3A–C). Co‐localization of mHTT and SIDT2 was rarely observed, as illustrated by immunofluorescence triple staining (Figure 3D).

### 3.3 SIDT2‐immunoreactive inclusions are present in an HD case with Vonsattel grade 0

**FIGURE 4:**
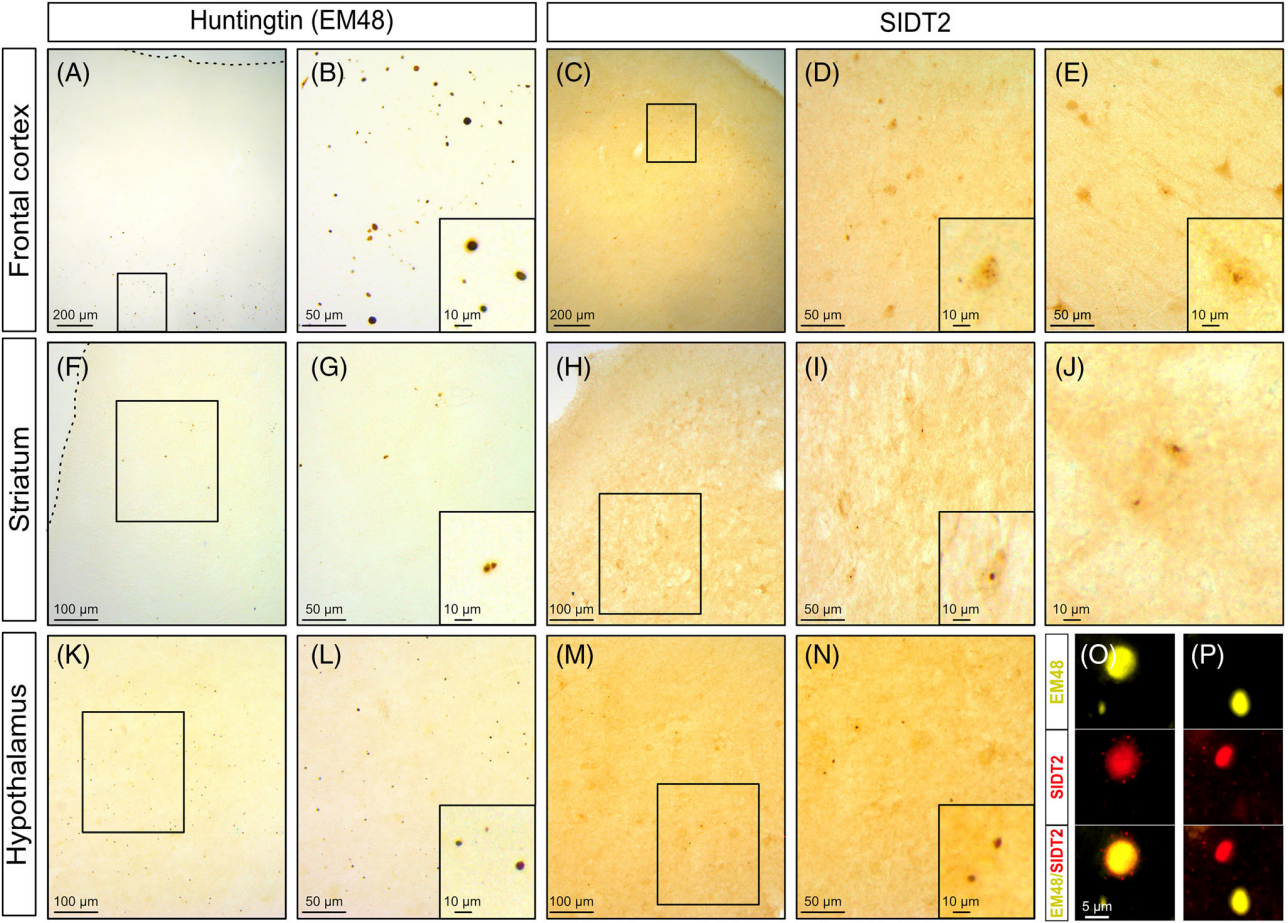
EM48‐ and SIDT2‐immunopositive inclusions in human postmortem tissue from an HD case with Vonsattel grade 0. EM48‐immunoreactive HTT inclusions are observed in the frontal cortex (A, B), the caudate nucleus of the striatum (F, G), and in the lateral hypothalamic area (K, L) of an HD brain with Vonsattel grade 0 (A). SIDT2‐immunoreactive inclusions are detected in the same brain regions (C–E, H–J, M, N). Immunofluorescent analysis shows that only a very few cells have co‐localization of SIDT2 and mHTT inclusions (O), whereas most SIDT2 inclusions do not co‐localize with mHTT inclusions (P), suggesting independent aggregation pathways (D).

To investigate the early presence of SIDT2 immunoreactivity as well as mHTT inclusions, we analyzed a rare HD case with 43 CAG repeats and a Vonsattel grade 0 [22]. We observed frequent EM48‐immunoreactive mHTT inclusions in the frontal cortex (Figure 4A,B), the caudate nucleus of the striatum (Figure 4F,G), and in the lateral hypothalamic area (Figure 4K,L). SIDT2 immunoreactive inclusions were detected in the same brain regions (Figure 4C–E, H–J, M, N). In the frontal cortex, SIDT2‐immunoreactive inclusions were mainly formed in layer II and V‐VI (Figure 4 C–E). Immunofluorescent analysis shows that only very few cells display co‐localization of SIDT2 and mHTT inclusions (Figure 4O) and that most SIDT2 inclusions do not co‐localize with mHTT inclusions (Figure 4P). This is consistent with observations in HD cases with higher Vonsattel grades (Figure 3D). Hence, most SIDT2‐immunoreactive inclusions do not co‐localize with EM48‐immunoreactive mHTT inclusions in postmortem human brain tissue from clinical HD cases.

### 3.4 SIDT2‐immunoreactive inclusions appear later than the formation of mHTT inclusions in the R6/2 HD mouse model

**FIGURE 5:**
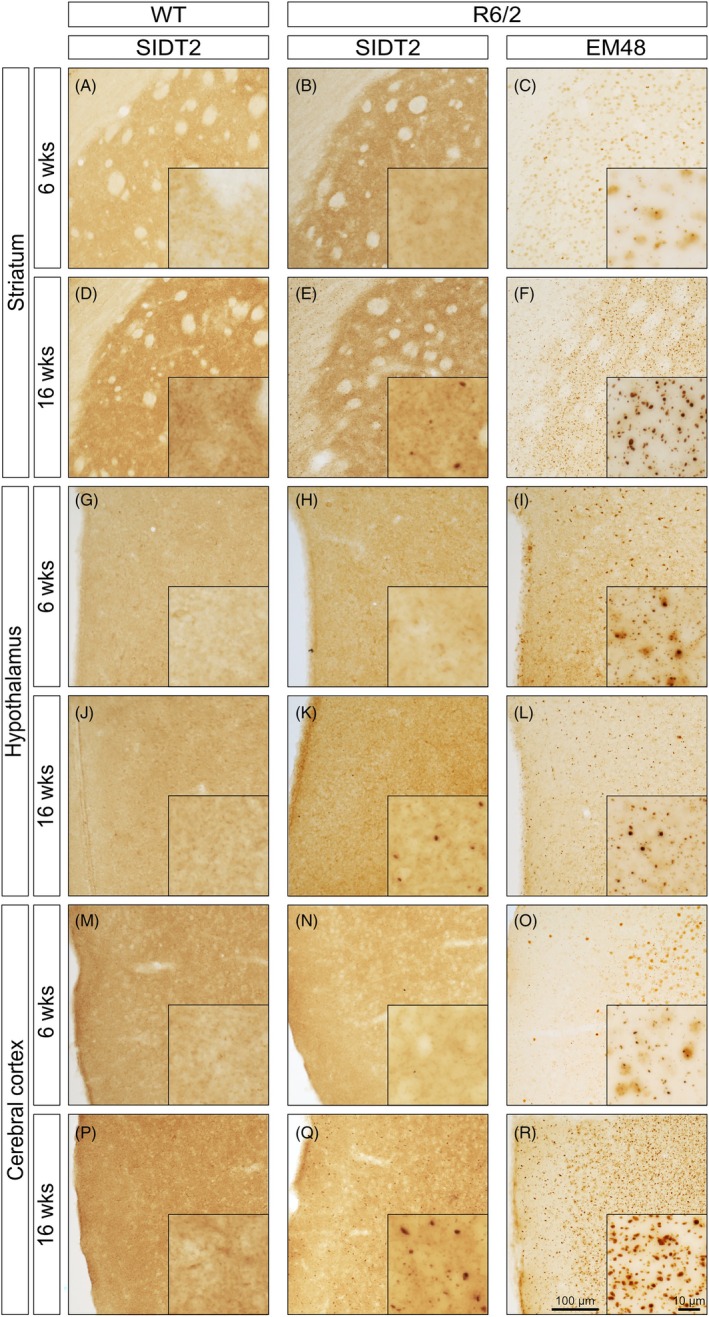
R6/2 mice form SIDT2‐immunoreactive inclusions following the formation of mHTT inclusions. In R6/2 mice at 6 weeks (wks) of age, single SIDT2‐immunoreactive inclusions are present in the cerebral cortex with no SIDT2 immunoreactivity in the striatum and hypothalamus (B, H, N). At this age, R6/2 mice display EM48‐immunoreactive mHTT inclusions in these brain regions (C, I, O). At 16 weeks of age, R6/2 mice have formed SIDT2‐immunoreactive inclusions in the striatum, hypothalamus, and cerebral cortex (E, K, Q) with an increased frequency of mHTT inclusions (F, J, R). Brain regions from wild‐type (WT) littermates of the same age show no SIDT2 or mHTT immunoreactivity (A, D, G, J, M, P).

**FIGURE 6:**
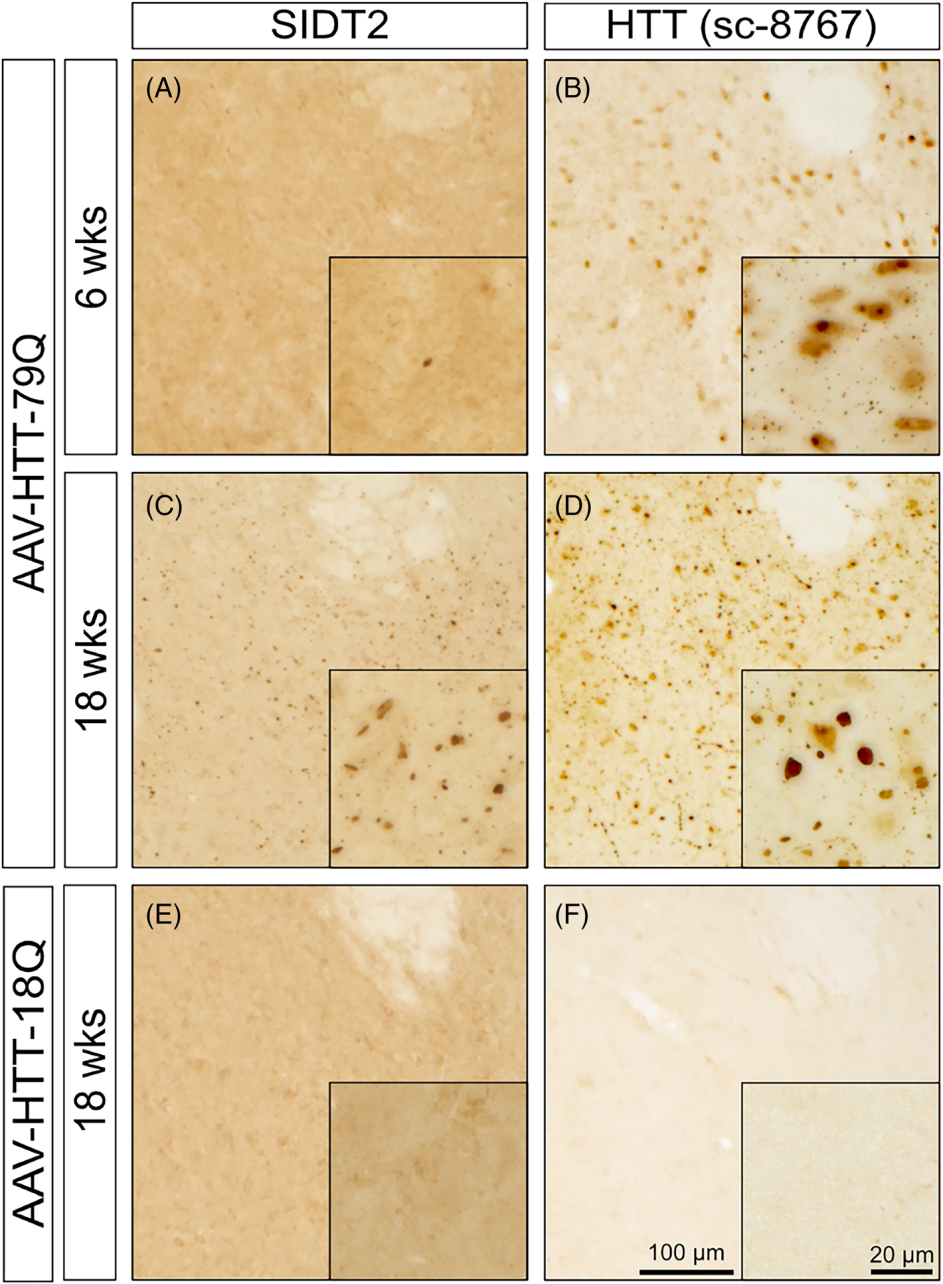
Time‐dependent formation of SIDT2 inclusions in the hypothalamus following AAV vector mediated mHTT overexpression. Presence of rare SIDT2‐immunoreactive inclusions and widespread mHTT inclusions in mice overexpressing mHTT in the hypothalamus following AAV‐HTT‐79Q injection at 6 weeks post‐injection (A, B). An increase in the frequency of SIDT2 inclusions and mHTT inclusions 18 weeks post‐injection of AAV‐HTT‐79Q (C, D). Control mice injected with AAV‐HTT‐18Q, expressing an 853 amino acid fragment of wild‐type huntingtin with 18Q, showed no detectable SIDT2 or mHTT immunoreactivity (E, F).

We then examined whether SIDT2‐immunoreactive inclusions were present in the brains of HD mouse models. In the R6/2 mouse model, we did not detect SIDT2‐immunoreactive inclusions at 6 weeks of age in the striatum, hypothalamus, and cerebral cortex (Figure 5B,H,N). However, at 16 weeks of age, SIDT2‐immunoreactive inclusions were present in these three regions in R6/2 mice (Figure 5E,K,Q). We did not detect any SIDT2‐immunoreactive inclusions in the striatum, the hypothalamus, or the cerebral cortex in any wild‐type littermates at these two time points (Figure 5A,D,G,J,M,P). As expected, EM48‐immunoreactive mHTT inclusions were present in these three brain regions at 6 weeks of age and showed a higher density at 16 weeks of age (Figure 5C,F,I,L,O,R). Based on the analyses of R6/2 brain tissues, SIDT2‐immunoreactive inclusions appear at a later stage than EM48‐immunoreactive mHTT inclusions. A similar observation was made in hypothalamic tissue of mice with AAV vector mediated overexpression of a large fragment of mHTT with 79 glutamines, where many cells formed mHTT inclusions at 6 weeks post‐injection, as reported previously [31], but only a few cells displayed SIDT2‐immunopositive inclusions (Figure 6A,B). At 18 weeks post‐injection, the density of mHTT inclusions was increased, as well as the density of SIDT2‐immunoreactive inclusions (Figure 6C,D). SIDT2‐immunoreactive inclusions or mHTT inclusions were not present after overexpression of a similar HTT fragment with a normal length of 18 glutamines at 18 weeks post‐injection (Figure 6E,F). We then examined SIDT2‐immunoreactivity in these brain regions in the BACHD mice expressing full‐length mHTT with 97 glutamines [29]. We could not detect any SIDT2‐ or EM48‐immunoreactive mHTT inclusions in the striatum, cerebral cortex, and hypothalamus of BACHD mice at 6 months of age, a stage when these mice display an overt phenotype (Figure S5). Taken together, SIDT2‐immunoreactive inclusions appear to develop after the formation of mHTT inclusions in HD mice.

### 3.5 Overexpression of SIDT2 using AAV vectors reduces the number of mHTT inclusions in the lateral hypothalamic area in R6/2 mice

**FIGURE 7:**
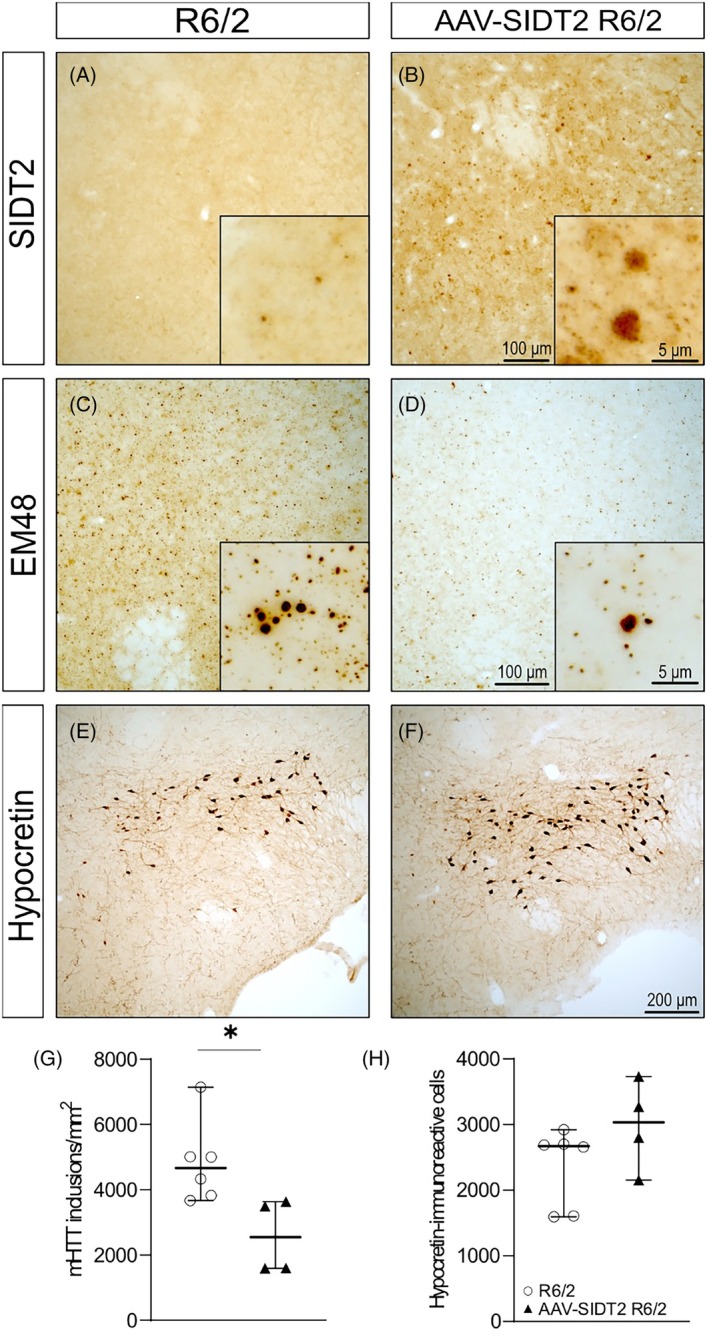
Overexpression of SIDT2 using AAV vectors reduces the number of mHTT inclusions in the hypothalamus in R6/2 mice. Immunohistochemistry for SIDT2 shows increased immunoreactivity in AAV‐SIDT2‐injected R6/2 mice at 10 weeks post‐injection compared to uninjected R6/2 controls of the same age (16 weeks of age; A, B). mHTT immunoreactivity is reduced in AAV‐SIDT2‐injected R6/2 mice compared to R6/2 controls (C, D), with a significant decrease in the density of mHTT inclusions in the lateral hypothalamic area in mice overexpressing SIDT2 (G; *p* = 0.010). While hypocretin‐immunoreactive neurons are decreased in R6/2 mice as reported in previous studies [37], mice overexpressing SIDT2 in the hypothalamus show a non‐significant trend towards increased hypocretin‐immunoreactive neurons (E, F, H; *p* = 0.171). The data are presented as median with range. Two‐tailed, Mann–Whitney *U* test, * *p* < 0.05.

Given that a previous study [20] demonstrated a reduction in mHTT inclusions following SIDT2 overexpression in vitro, we conducted an in vivo proof‐of‐principle experiment. We designed an AAV vector expressing human SIDT2 that was injected into the hypothalamus of R6/2 mice at 6 weeks of age, and the effects were assessed 10 weeks post‐injection. We confirmed the presence of AAV vector delivered SIDT2 by immunohistochemistry using an antibody against SIDT2 (Figure 7A,B). The R6/2 mice, similarly to persons with HD, show loss of hypocretin (orexin) neurons in the lateral hypothalamic area [5, 37]. Therefore, we examined the lateral hypothalamic area and found that AAV‐mediated overexpression of SIDT2 resulted in a 45% reduction in the density of mHTT inclusions (*p* = 0.01) and a trend towards a 16% increase in hypocretin‐immunoreactive neurons compared to uninjected R6/2 mice at the same age (*p* = 0.17). There were no significant differences in body weight between SIDT2‐overexpressing R6/2 mice compared to uninjected R6/2 mice (data not shown).

### 3.6 Overexpression of SIDT2 reduces soluble and insoluble mHTT protein in SH‐SY5Y cells without affecting major proteolytic degradation pathways

**FIGURE 8:**
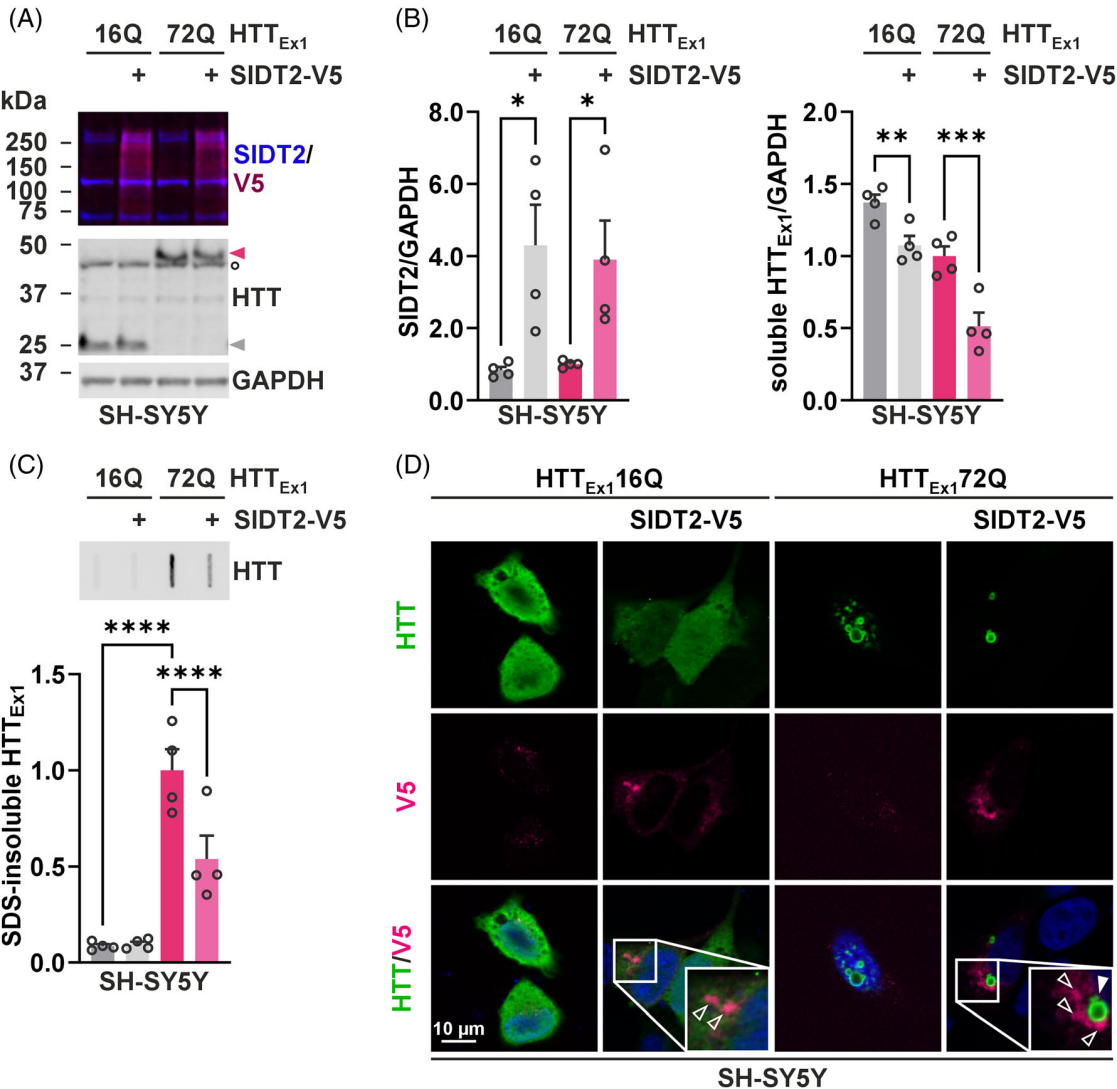
Overexpression of SIDT2 lowers soluble and SDS‐insoluble forms of HTT exon 1 in SH‐SY5Y cells. Protein extracts from SH‐SY5Y cells co‐transfected with HTT exon 1 (HTT_Ex1_) with 16Q or 72Q, along with V5‐tagged SIDT2 or an empty control vector, were analyzed by Western blotting and filter retardation assay. (A) Membranes were incubated with antibodies specific for SIDT2, the V5 tag, and HTT. GAPDH served as the loading control. The gray arrowhead marks HTT_Ex1_ 16Q, while the magenta arrowhead indicates HTT_Ex1_ 72Q. The black circle denotes an HTT_Ex1_‐unspecific band. (B) Quantitative analysis confirmed the overexpression of SIDT2 and demonstrated a significant reduction in soluble HTT_Ex1_ 16Q and 72Q levels upon SIDT2 overexpression. Signals were normalized to the loading control and then to the reference condition (HTT_Ex1_ 72Q/ empty). Bars represent mean + S.E.M. *n* = 4. *p ≤ 0.05, **p ≤ 0.01, ***p ≤ 0.001 (Two‐way ANOVA with Fisher's LSD test). (C) Filter retardation assay of SH‐SY5Y cells co‐transfected with HTT_Ex1_ with 16Q or 72Q with V5‐tagged SIDT2 or an empty control vector. HTT was detected using an HTT‐specific antibody, and densitometric analysis demonstrated a significant SIDT2‐dependent reduction of SDS‐insoluble HTT_Ex1_ 72Q. Signals were normalized to the respective control condition (HTT_Ex1_ 72Q/empty). Bars represent mean + S.E.M. *n* = 4. ****p ≤ 0.0001 (Two‐way ANOVA with Fisher's LSD test). (D) Immunocytochemistry staining and fluorescence microscopy of SH‐SY5Y cells co‐transfected with HTT_Ex1_ with 16Q or 72Q, along with V5‐tagged SIDT2 or an empty control vector. HTT was detected using an HTT‐specific antibody (green), while overexpressed SIDT2 was stained with V5 tag‐specific antibody (red). Overexpressed SIDT2 formed inclusion‐like structures (white‐rimmed arrowheads), which did not co‐localize with HTT_Ex1_ 72Q inclusion bodies (solid white arrowheads). DAPI was used as a nuclear counterstain (blue). Insets show 2‐fold magnifications of selected areas (white boxes). Bars represent mean + S.E.M. *n* = 4. *p ≤ 0.05, **p ≤ 0.01, ***p ≤ 0.001, ****p ≤ 0.0001 (Two‐way ANOVA with Fisher's LSD test).

A previous study demonstrated that SIDT2 overexpression leads to a reduction in *HTT* mRNA, as well as reduced soluble and insoluble mHTT protein levels in murine Neuro2a cells co‐transfected with vectors encoding EGFP‐tagged *HTT* exon 1 and mouse *Sidt2* [20]. To validate both our observations in R6/2 mice and these earlier findings in mouse cells in a human cell model system, we replicated the corresponding condition in SH‐SY5Y neuroblastoma cells overexpressing untagged *HTT* exon 1 16Q and 72Q along with human V5‐tagged *SIDT2*. Western blot analysis confirmed an approximately 4‐fold overexpression of SIDT2 resulted in a significant reduction of soluble HTT exon 1 16Q and, even more pronounced, polyQ‐expanded HTT exon 1 72Q (Figure 8A,B). To assess potential effects on HTT exon 1 72Q aggregation, we performed filter retardation assays to detect SDS‐insoluble HTT. Consistent with our findings on soluble HTT exon 1, insoluble HTT exon 1 72Q levels was significantly reduced upon SIDT2 overexpression (Figure 8C).

Since SIDT2 has been reported to not only modulate RNautophagy but also macroautophagy in general [38], we sought to determine whether the observed effects on soluble and insoluble HTT exon 1 levels were due to changes in baseline autophagy. To test this and to investigate potential effects on the UPS, we analyzed protein markers of both pathways. Western blot analysis revealed no significant changes in K48‐linked polyubiquitinated proteins (a marker for the UPS), SQSTM1/p62 levels, or the LC3B‐II to LC3B‐I ratio, indicating no alterations in baseline autophagy (Figure S6A,B). These findings rule out the activation of either proteolytic degradation pathway as the underlying cause of SIDT2‐driven HTT exon 1 reduction in our cell model.

Given our previous observations of SIDT2‐immunoreactive inclusions in both HD patient brain tissue and HD mice, we extended our microscopy analysis to the SH‐SY5Y overexpression model. Epi‐fluorescence microscopy confirmed the presence of inclusion‐like structures in cells overexpressing SIDT2, which did not directly co‐localize with HTT exon 1 72Q inclusion bodies (Figure 8D). In summary, our human neuroblastoma cell‐based analysis confirms previous reports and our in vivo findings, demonstrating that SIDT2 overexpression lowers both soluble and insoluble mHTT levels. Additionally, our results support the potential of SIDT2 to form inclusion‐like structures.

## 4 DISCUSSION

SIDT2 is a lysosomal protein involved in RNA and DNA autophagy. Here we provide the first evidence of significant alterations of the SIDT2 system in postmortem brain tissue from persons with HD and in multiple HD models. We show that SIDT2‐immunoreactive inclusions are present in brain tissue displaying mHTT inclusions, although they seldom co‐localize. Inclusions with SIDT2 are readily detected in the caudate nucleus of the striatum, the frontal cortex, and the lateral hypothalamic area in human postmortem HD tissue as well as in animal models of HD. Moreover, SIDT2‐immunoreactive inclusions appear to form in a CAG‐repeat dependent fashion subsequent to the formation of mHTT inclusions. At later HD stages, SIDT2 protein levels are significantly reduced, particularly in the striatum but also in the lateral hypothalamic area, without changes in *SIDT2* mRNA levels. This suggests a potential depletion of soluble SIDT2 during attempted mHTT degradation. Interestingly, consistent with a prior in vitro study [20], we show a significant reduction of mHTT load after SIDT2 overexpression in both R6/2 mice and a neuroblastoma cell line overexpressing mHTT exon 1. Taken together, our data demonstrate novel neuropathological features involving SIDT2 in HD and further support the potential therapeutic effects of modulating this pathway.

Neuropathological hallmarks in HD include the presence of mHTT containing inclusions. The present study reveals that SIDT2‐immunoreactive inclusions constitute an additional neuropathological hallmark. Since SIDT2‐immunoreactive inclusions rarely co‐localized with mHTT inclusions, as assessed with the EM48‐antibody, these inclusions may occur independently. However, our analyses indicate that SIDT2 inclusions only form in brain tissue that also contains mHTT inclusions, although at a later stage. Therefore, the formation of SIDT2 inclusions and mHTT inclusions may reflect similar underlying pathogenic processes occurring in parallel.

To our knowledge, only one prior published study has investigated SIDT2 in HD [20]. That study showed that SIDT2 binds to *HTT* mRNA in a CAG dependent fashion. Here we detected the formation of SIDT2‐immunoreactive inclusions in affected HD brain tissues and observed an increased density of SIDT2 inclusions in HD cases with higher CAG repeats in the ACC. Both previous and recent studies have shown somatic expansion of the *mHTT* gene in the striatum and cerebral cortex in postmortem cases with HD [39, 40, 41, 42]. It is possible that the SIDT2‐containing inclusions also reflect the presence of somatic expansion in *mHTT* RNA. As SIDT2 binds to CAG repeats, the formation of SIDT2 inclusions may also be present in brains from persons with other CAG triplet repeat disorders such as the spinocerebellar ataxias. It would therefore be interesting to examine whether SIDT2‐immunoreactive inclusions are present in brains from persons with such disorders.

This study demonstrates for the first time that overexpression of SIDT2 can reduce the load of mHTT inclusions in vivo. Our proof‐of‐principle experiment using AAV vectors to overexpress SIDT2 led to a significant reduction of mHTT inclusions in the lateral hypothalamic area of R6/2 mice and trends towards beneficial effects on hypocretin neuronal loss. Further studies examining effects in a larger cohort of mice including in other HD animal models and in other affected brain regions with different concentrations of the AAV vector will be important to evaluate the full potential of SIDT2 overexpression on HD pathology. Moreover, in the present study we also demonstrate that overexpression of human SIDT2 in human SH‐SY5Y neuroblastoma cells leads to a reduction in both soluble and insoluble levels of untagged HTT exon 1, confirming findings from a murine cell model employing human GFP‐tagged HTT exon 1 constructs [20]. Interestingly, SIDT2 overexpression alone resulted in the formation of inclusion‐like structures that did not directly co‐localize with inclusion bodies formed by polyQ‐expanded HTT exon 1 72Q, mirroring, to a certain degree, our observations in HD patient and R6/2 mouse brains.

Since SIDT2 has been implicated in macroautophagy, including mitophagy [38, 43, 44, 45], we analyzed baseline levels of the autophagy markers SQSTM1/p62 and LC3B but did not detect any modulatory effects upon SIDT2 overexpression. This suggests that the observed HTT reduction does not occur outside the context of RNautophagy. Additionally, our data showing unchanged K48‐polyubiquitin levels suggest that SIDT2 does not impact the UPS. However, further investigations into a potential link between SIDT2 and macroautophagy, which is known to be dysregulated in HD and contributes to HTT clearance [46], are necessary to fully rule out additional regulatory effects.

Moreover, while incomplete splicing‐derived HTT exon 1 is considered a primary contributor to HD pathology [47, 48, 49], and targeting this protein species or its coding RNA via SIDT2 appears to be an effective approach, further research into the effects on full‐length HTT mRNA and protein may provide insights into molecular interactions relevant to HD pathology. Additionally, exploring potential effects of other RNautophagy/uptake‐linked proteins such as LAMP2C and SIDT1 [14, 50, 51] on *HTT* mRNA may be helpful to further elucidate and optimize this strategy for HTT lowering.

Lysosomal pathways have been implicated in other neurodegenerative disorders including synucleinopathies. In fact, SIDT2‐immunoreactive inclusions have been described in the ACC of persons with these disorders [52]. Furthermore, SIDT2 may exert other important roles in the cell [53]. Recent studies have shown that SIDT2 can be localized to the plasma membrane and mediate the uptake of single and double‐stranded oligonucleotides in cells [54, 55, 56]. This has relevance for the development of nucleic acid‐based therapies, including those attempted for HD using anti‐sense oligonucleotides (ASO) for reducing mHTT levels [1]. The mechanisms underlying the cellular uptake of such oligonucleotide therapeutics are unclear. Interestingly, a recent study showed that inducing SIDT2 expression in human cell lines increased knock‐down activity of gapmer ASOs [56]. It is therefore possible that reductions of SIDT2 levels, as demonstrated in the present study in the striatum in HD and/or SIDT2 inclusion formation as shown in several affected HD brain tissue, may have a negative impact on the uptake of ASOs into cells. Further studies on the impact of SIDT2 on uptake of oligonucleotide therapeutics for HD are therefore warranted.

In conclusion, we identified a novel neuropathological hallmark with the formation of SIDT2‐immunopositive inclusions in affected brain tissues in HD. Our results suggest that targeting SIDT2‐mediated RNautophagy may have beneficial effects on reducing the load of mHTT inclusions independent of traditional protein degradation pathways. Recent studies suggest that SIDT2 may play a role in the uptake of extracellular nucleotides. Combined with our data showing significantly reduced SIDT2 protein levels in the striatum of advanced HD cases, this indicates that alterations in the SIDT2 system could impact the development of nucleotide‐based therapeutics for HD.

## AUTHOR CONTRIBUTIONS

All authors contributed to the study conception and design. Material preparation, data collection, or data analyses were performed by all authors. The first draft of the manuscript was written by Sanaz Gabery, Sofia Bergh, and Åsa Petersén. All authors read and commented on previous versions of the manuscript. All authors read and approved the final manuscript.

## FUNDING INFORMATION

This study was funded by research grants to AP from the Swedish Research Council (grant number 2022/01092), the Swedish Brain Foundation, the Foundation of King Gustav V and Queen Victoria, the Swedish governmental funding of clinical research (ALF) at Region Skåne as well as the Knut and Alice Wallenberg Foundation (#2019.0467). HPN and JJW were funded by the German Research Foundation (DFG; research grant numbers NG 101/6‐1 and WE 6585/1‐1, respectively). GMH was funded by the National Health and Medical Research Council of Australia [Investigator Grant 1176607]. EPP was funded by the Deutsche Huntington Hilfe (RUB No. 4908100115).

## CONFLICT OF INTEREST STATEMENT

The authors report no conflicts of interest.

## ETHICS STATEMENT

Human postmortem tissues were obtained from the Victorian Brain Bank and the Sydney Brain Bank at Neuroscience Research, Australia after approvals from their Scientific Committees (PID073, PID0111, PID167). All persons had given their informed consent prior to the donation of their brains and the regional brain donor programs were approved by the Institutional Human Research Ethics Committees. The analyses of human postmortem tissue in Bochum were approved by the Ethic Committee of the Medical Faculty of the Ruhr‐University Bochum, Germany (Reg. No. 17‐5939). Analyses performed on human tissue in Sweden were approved by the Swedish Ethical Review Authority (reference number 2022‐05348‐01) and the region ethical review board at Lund University (reference number 2014/466). The experimental procedures performed in animals were approved by the Regional Ethical Committee in Lund, Sweden under permit number 17113/2022.

## Supporting information


**FIGURE S1.** Western blots of SIDT2 of striatal tissue from HD cases compared to age‐ and sex‐matched control cases.
**FIGURE S2.** Western blots of SIDT2 of striatal tissue from HD cases compared to age‐ and sex‐matched control cases.
**FIGURE S3.** Western blots of cortical tissue from HD cases compared to age‐ and sex‐matched control cases.
**FIGURE S4.** Western blots of hypothalamic tissue from HD cases compared to age‐ and sex‐matched control cases.
**FIGURE S5.** Evaluation of SIDT2 and EM48 immunoreactivity in the BACHD mice.
**FIGURE S6.** No alterations in UPS‐ and autophagy‐related markers in SH‐SY5Y cells co‐expressing HTT exon 1 and SIDT2.
**TABLE S1.** Primary antibodies used for Western blot analyses of cell cultures.

## Data Availability

The data that support the findings of this study are available from the corresponding author upon reasonable request.
