# Supplementary material for: The DNA/RNA autophagy protein SIDT2 as a novel neuropathological hallmark in Huntington disease
Source: Brain Pathol. 2026 Feb 24;36(5):e70088. doi: 10.1111/bpa.70088 (PMC13429292; doi:10.1111/bpa.70088)
Supplement: Supplementary file 1 — FIGURE S1. Western blots of SIDT2 of striatal tissue from HD cases compared to age‐ and sex‐matched control cases. FIGURE S2. Western blots of SIDT2 of striatal tissue from HD cases compared to age‐ and sex‐matched control cases. FIGURE S3. Western blots of cortical tissue from HD cases compared to age‐ and sex‐matched control cases. FIGURE S4. Western blots of hypothalamic tissue from HD cases compared to age‐ and sex‐matched control cases. FIGURE S5. Evaluation of SIDT2 and EM48 immunoreactivity in the BACHD mice. FIGURE S6. No alterations in UPS‐ and autophagy‐related markers in SH‐SY5Y cells co‐expressing HTT exon 1 and SIDT2. TABLE S1. Primary antibodies used for Western blot analyses of cell cultures. [file BPA-36-e70088-s001.docx]

**Supplementary information:**

**The DNA/RNA autophagy protein SIDT2 as a novel neuropathological hallmark**

**in Huntington disease**

Sanaz Gabery^1*^, Sofia Bergh^1*^, Chrisovalantou Huridou^2,3^, Rachel Y Cheong^1^ , Barbara Baldo^1^, Paul Günther Scheunemann^4^, Marie-Louisa Schoebel^4^, Linda Holmquist Mengelbier^1^, Elisabet Englund^5^, Catriona McLean^6^, Carsten Saft^7,8^, Deniz Kirik^9^, Maria Björkqvist^10+^, Glenda Halliday^11^, Elisabeth Petrasch-Parwez^4^, Huu Phuc Nguyen^2,8^, Jonasz J. Weber^2,3^, Åsa Petersén^1, 12^

^1^Translational Neuroendocrine Research Unit, Department of Experimental Medical Science, Lund University, Lund, Sweden

^2^Department of Human Genetics, Medical Faculty, Ruhr University Bochum, Bochum, Germany

^3^Institute of Medical Genetics and Applied Genomics, University of Tübingen, Tübingen, Germany

^4^Department of Neuroanatomy and Molecular Brain Research, Medical Faculty,Ruhr University Bochum, Bochum, Germany

^5^ Division of Pathology, Department of Clinical Sciences, Lund University, Lund Sweden

^6^Department of Pathology, Alfred Hospital, Melbourne, Victoria, Australia

^7^Department of Neurology, Huntington Centre NRW, St. Josef-Hospital, Ruhr-University Bochum, Bochum, Germany

^8^Huntington Center NRW, Ruhr-University Bochum, Bochum, Germany

^9^Deniz affiliation, Department of Experimental Medical Science, Lund University, Lund, Sweden

^10^Brain Disease Biomarker Unit, Department of Experimental Medical Science, Lund University, Lund, Sweden

^11^The Brain and Mind Centre and Faculty of Medicine and Health, School of Medical Sciences, University of Sydney, Sydney, Australia

^12^Department of Psychiatry, Skåne University Hospital, Lund, Sweden

*shared first authors

Sanaz Gabery: [sanazgabery85@gmail.com](mailto:sanazgabery85@gmail.com)
Sofia Bergh: [sofia.bergh@med.lu.se](mailto:sofia.bergh@med.lu.se)
Chrisovalantou Huridou: [Chrisovalantou.Huridou@med.uni-tuebingen.de](mailto:Chrisovalantou.Huridou@med.uni-tuebingen.de)
Rachel Y Cheong: [ry.cheong@gmail.com](mailto:ry.cheong@gmail.com)
Barbara Baldo: [barbara.baldo83@gmail.com](mailto:barbara.baldo83@gmail.com)
Paul Günther Scheunemann: [paul.scheunemann@ruhr-uni-bochum.de](mailto:paul.scheunemann@ruhr-uni-bochum.de)
Marie-Louisa Schoebel: Marie-Louisa.Schoebel@ruhr-uni-bochum.de
Linda Holmquist Mengelbier: [linda.holmquist_mengelbier@med.lu.se](mailto:linda.holmquist_mengelbier@med.lu.se)
Elisabet Englund: [elisabet.englund@med.lu.se](mailto:elisabet.englund@med.lu.se)
Catriona McLean: [C.McLean@alfred.org.au](mailto:C.McLean@alfred.org.au)
Carsten Saft: [carsten.saft@ruhr-uni-bochum.de](mailto:carsten.saft@ruhr-uni-bochum.de)
Deniz Kirik: [deniz.kirik@med.lu.se](mailto:deniz.kirik@med.lu.se)
Maria Björkqvist: [maria.bjorkqvist@med.lu.se](mailto:maria.bjorkqvist@med.lu.se)
Glenda Halliday: [glenda.halliday@sydney.edu.au](mailto:glenda.halliday@sydney.edu.au)
Elisabeth Petrasch-Parwez:[elisabeth.petrasch-parwez@ruhr-uni-bochum.de](mailto:elisabeth.petrasch-parwez@ruhr-uni-bochum.de)
Huu Phuc Nguyen: [huu.nguyen-r7w@rub.de](mailto:huu.nguyen-r7w@rub.de)
Jonasz J. Weber: [Jonasz.Weber@med.uni-tuebingen.de](mailto:Jonasz.Weber@med.uni-tuebingen.de)
Åsa Petersén: [asa.petersen@med.lu.se](mailto:asa.petersen@med.lu.se)

**Corresponding author:** Professor Åsa Petersén MD PhD, Translational Neuroendocrine Research Unit, Department of Experimental Medical Science, Lund University, BMC D11, 221 84 Lund, Sweden. E-mail: asa.petersen@med.lu.se

Phone: + 46 46 2221686

**Running title:** SIDT2 in Huntington disease

**Supplementary Figure legends**

**Supplementary Figure 1. Western blots of SIDT2 of striatal tissue from HD cases compared to age- and sex-matched control cases.**

Original Western blots of the caudate nucleus of the striatum using Antibody 1 (Abcam ab85847 antibody) with protein ladder.

**Supplementary Figure 2. Western blots of SIDT2 of striatal tissue from HD cases compared to age- and sex-matched control cases.**

Original Western blots of the caudate nucleus of the striatum using Antibody 2 (Abnova PAB27211) with protein ladder.

**Supplementary Figure 3. Western blots of cortical tissue from HD cases compared to age- and sex-matched control cases.**

Original Western blots of frontal cortical tissue using Antibody 1 (Abcam ab85847 antibody) with protein ladder.

**Supplementary Figure 4. Western blots of hypothalamic tissue from HD cases compared to age- and sex-matched control cases.**

Original Western blots of tissue from the lateral hypothalamic area using Antibody 2 (Abnova PAB27211) with protein ladder.

**Supplementary Figure 5. Evaluation of SIDT2 and EM48 immunoreactivity in the BACHD mice.**

No SIDT2-immunoreactive and EM48-immunoreactive inclusions were detected in the striatum, cerebral cortex, or hypothalamus of 6 month old BACHD mice, a stage characterized by an overt phenotype.

**Supplementary Figure 6. No alterations in UPS- and autophagy-related markers in SH-SY5Y cells co-expressing HTT exon 1 and SIDT2.**

Protein extracts from SH-SY5Y cells co-transfected with HTT exon 1 (HTT_Ex1_) with 16Q or 72Q, along with V5-tagged SIDT2 or an empty control vector, were analyzed by Western blotting to assess alterations in ubiquitin-proteasome system (UPS) and autophagy. (**a**) Membranes were incubated with antibodies specific for K48-linked polyubiquitin (K48-pUb) (UPS), as well as SQSTM1/p62 (p62), and LC3B (both autophagy markers). GAPDH served as the loading control. Black arrowheads indicate unmodified LC3B-I (I) and lapidated LC3B-II (II). (**b**) Quantitative analysis did not show any baseline-variation in UPS- and autophagy-related markers upon HTT_Ex1_ 72Q or SIDT2 overexpression. K48-pUb and p62 signals were normalized to the loading control and then to the reference condition (HTT_Ex1_ 72Q/empty vector), while LC3B signals are presented as the LC3B-II/LC3B-I ratio. *n* = 4. Bars represent means ± s.e.m.

# Supplementary Figures

# Supplementary figure 1


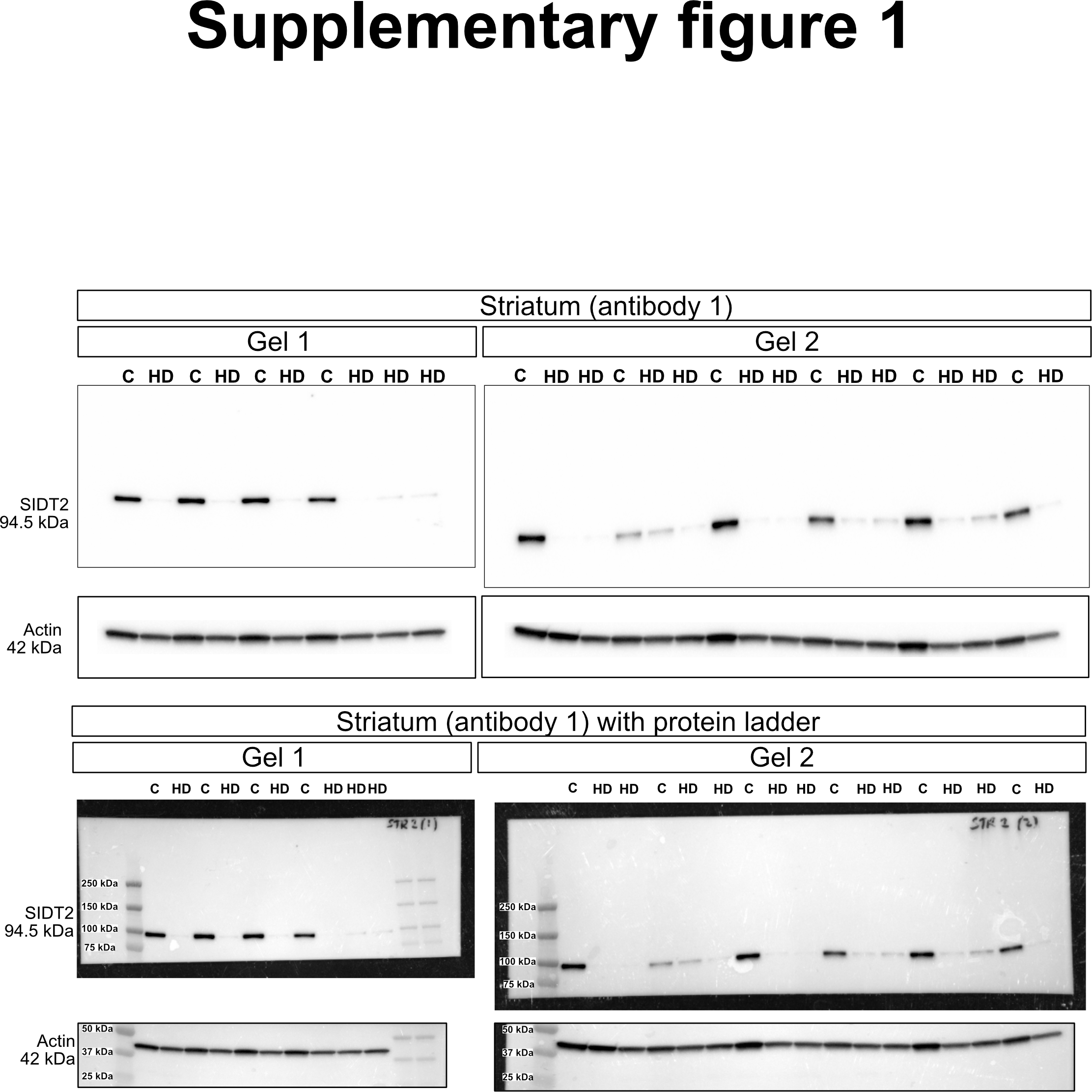


**Supplementary Figure 1. Western blots of SIDT2 of striatal tissue from HD cases compared to age- and sex-matched control cases.**

Original Western blots of the caudate nucleus of the striatum using Antibody 1 (Abcam ab85847 antibody) with protein ladder.

# Supplementary figure 2


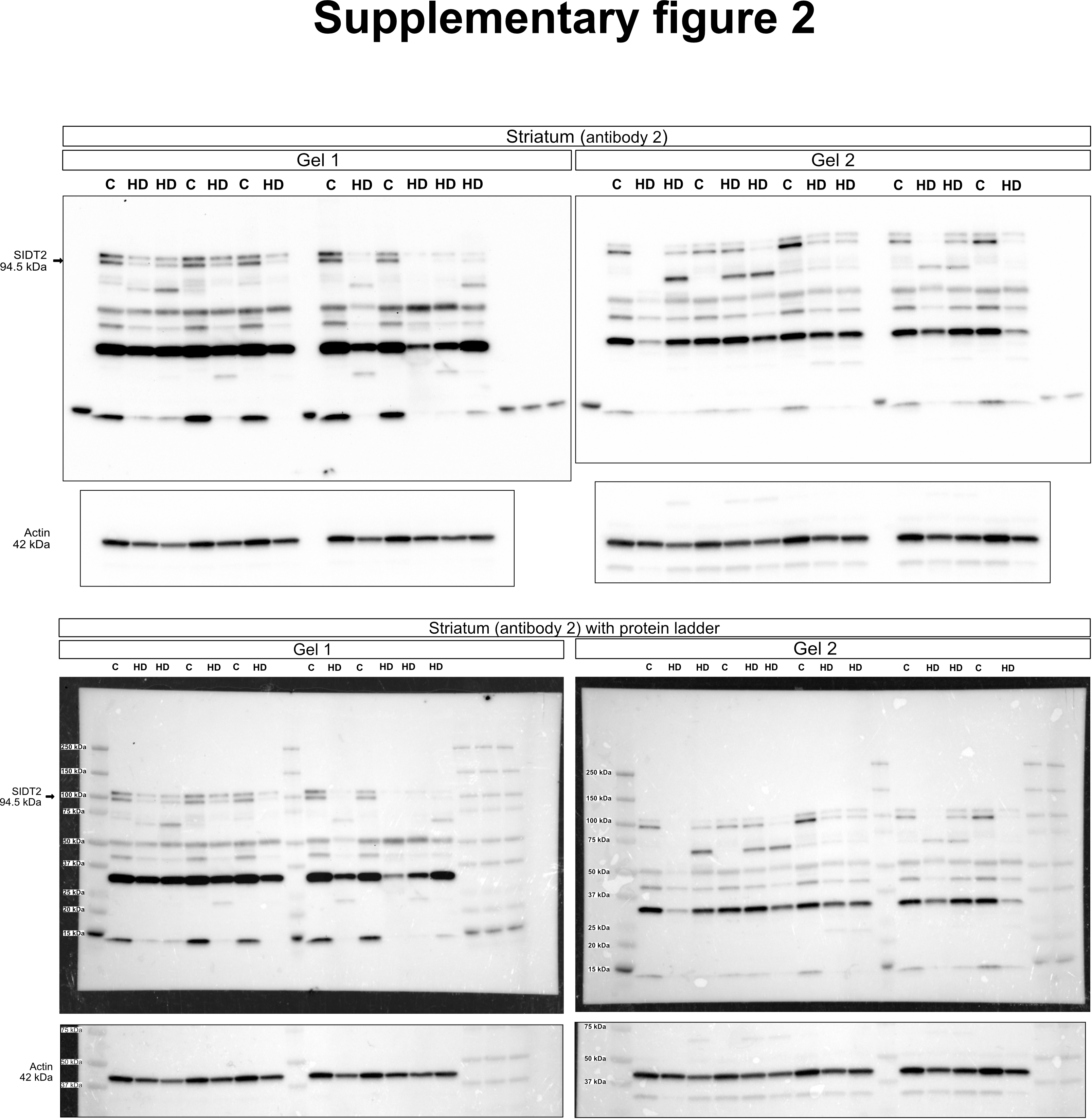


**Supplementary Figure 2. Western blots of SIDT2 of striatal tissue from HD cases compared to age- and sex-matched control cases.**

Original Western blots of the caudate nucleus of the striatum using Antibody 2 (Abnova PAB27211) with protein ladder.

# Supplementary figure 3


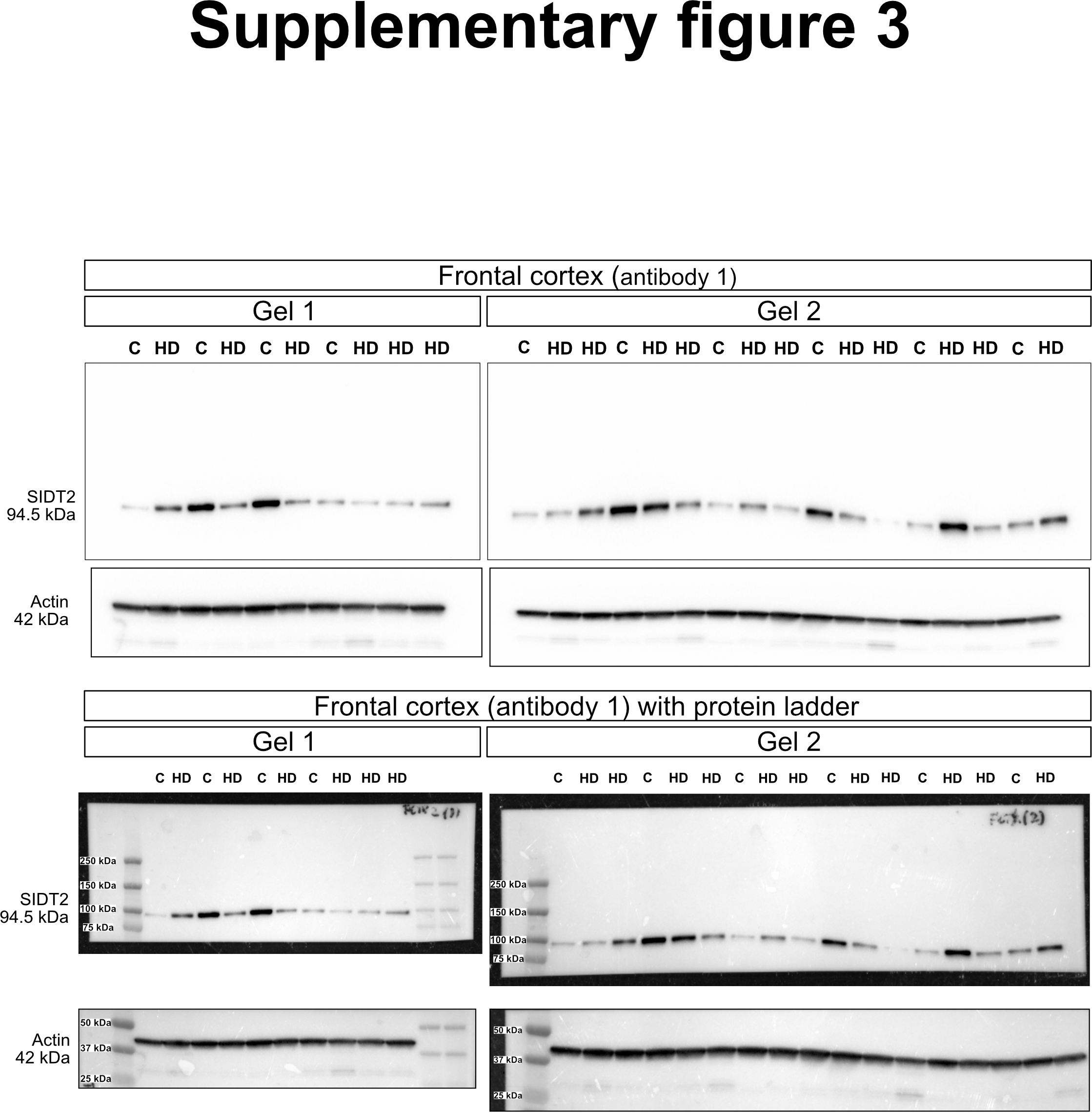


**Supplementary Figure 3. Western blots of cortical tissue from HD cases compared to age- and sex-matched control cases.**

Original Western blots of frontal cortical tissue using Antibody 1 (Abcam ab85847 antibody) with protein ladder.

# Supplementary figure 4


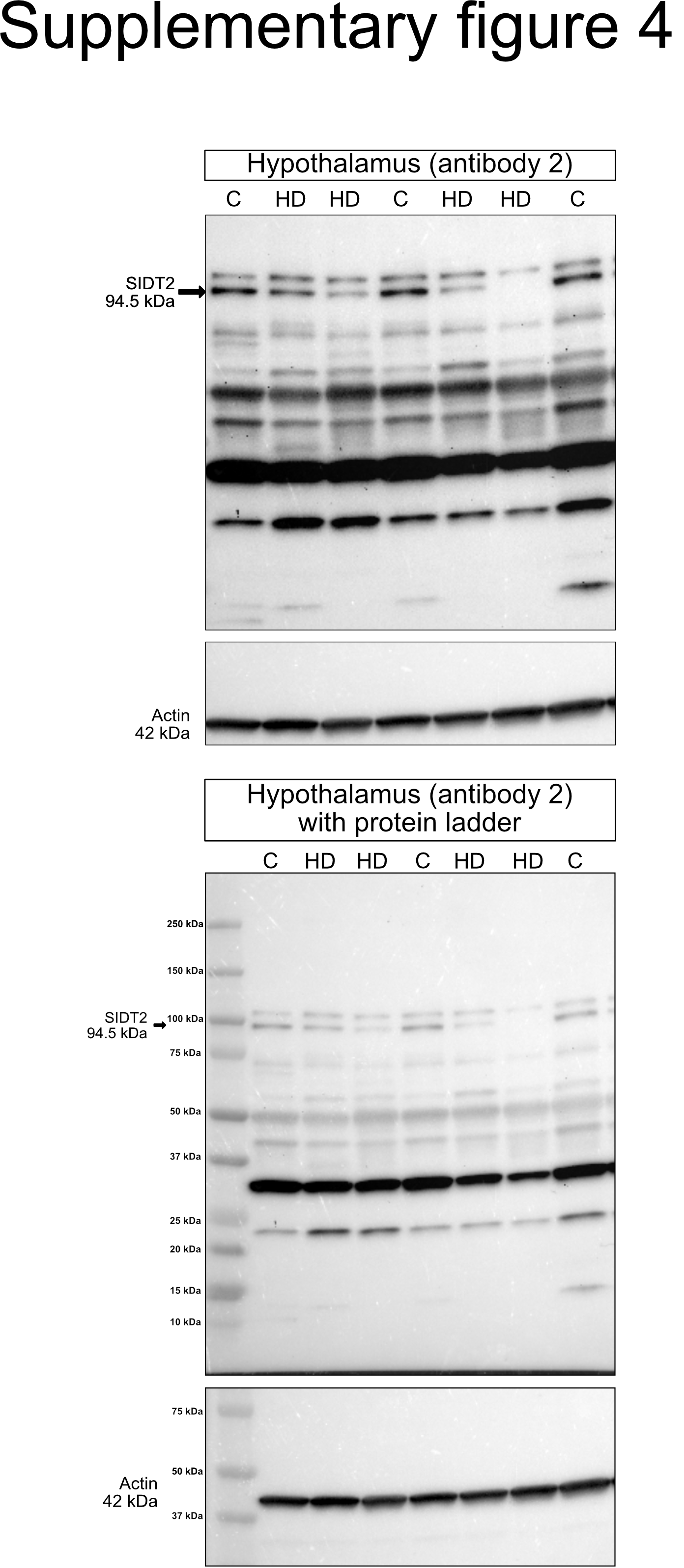


**Supplementary Figure 4. Western blots of hypothalamic tissue from HD cases compared to age- and sex-matched control cases.**

Original Western blots of tissue from the lateral hypothalamic area using Antibody 2 (Abnova PAB27211) with protein ladder.

**Supplementary figure 5**

**Supplementary Figure 5. Evaluation of SIDT2 and EM48 immunoreactivity in the BACHD mice.**

No SIDT2-immunoreactive and EM48-immunoreactive inclusions were detected in the striatum, cerebral cortex, or hypothalamus of 6 month old BACHD mice, a stage characterized by an overt phenotype.

**Supplementary figure 6**


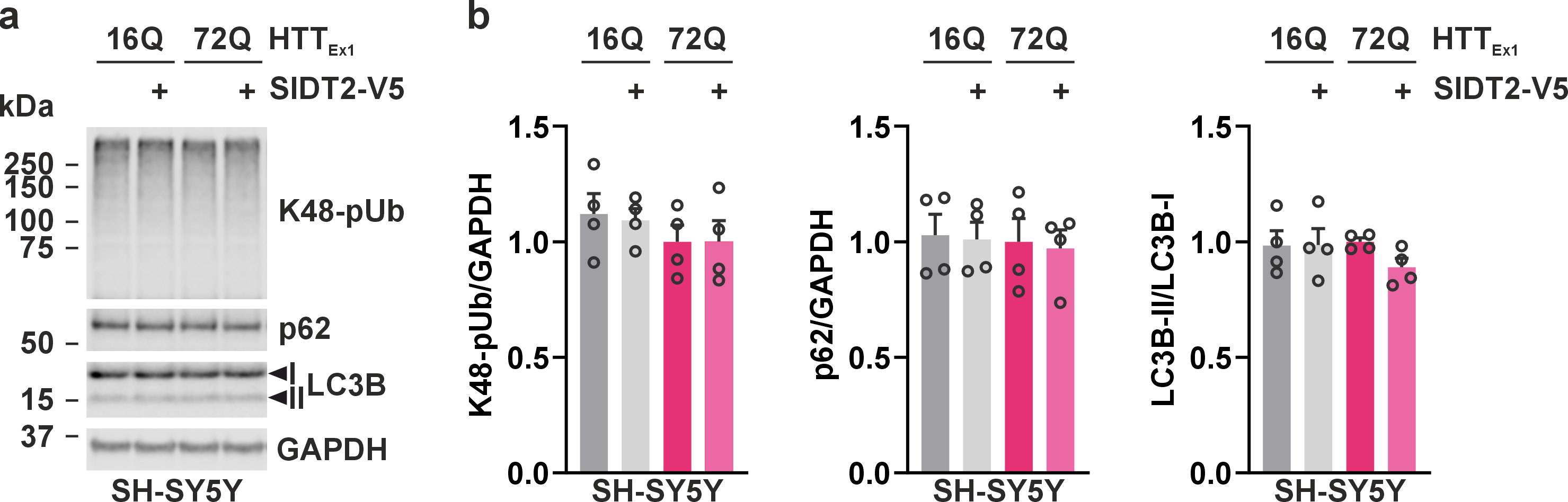


**Supplementary Figure 6. No alterations in UPS- and autophagy-related markers in SH-SY5Y cells co-expressing HTT exon 1 and SIDT2.**

Protein extracts from SH-SY5Y cells co-transfected with HTT exon 1 (HTT_Ex1_) with 16Q or 72Q, along with V5-tagged SIDT2 or an empty control vector, were analyzed by Western blotting to assess alterations in ubiquitin-proteasome system (UPS) and autophagy. (**a**) Membranes were incubated with antibodies specific for K48-linked polyubiquitin (K48-pUb) (UPS), as well as SQSTM1/p62 (p62), and LC3B (both autophagy markers). GAPDH served as the loading control. Black arrowheads indicate unmodified LC3B-I (I) and lapidated LC3B-II (II). (**b**) Quantitative analysis did not show any baseline-variation in UPS- and autophagy-related markers upon HTT_Ex1_ 72Q or SIDT2 overexpression. K48-pUb and p62 signals were normalized to the loading control and then to the reference condition (HTT_Ex1_ 72Q/empty vector), while LC3B signals are presented as the LC3B-II/LC3B-I ratio. *n* = 4. Bars represent means ± s.e.m.

**Supplementary Table**

**Supplementary Table S1: Primary antibodies used for Western blot analyses of cell cultures**

| **Antibody/target** | **Host** | **Dilution** | **Clone/catalogue no.** | **Vendor/source** |
| --- | --- | --- | --- | --- |
| β-actin | mouse | 1:5,000 | AC-15/A5441 | Sigma-Aldrich |
| GAPDH | mouse | 1:2,000 | 0411/sc-47724 | Santa Cruz |
| GAPDH | rabbit | 1:5,000 | 10494-1-AP | Proteintech |
| HTT | rabbit | 1:5,000 | EPR5526/ab109115 | Abcam |
| LC3B | rabbit | 1:500* | #2775 | Cell Signaling |
| p62/SQSTM1 | rabbit | 1:500* | #5114 | Cell Signaling |
| SIDT2 | rabbit | 1:1,1000 | PAB27211 | Abnova |
| ubiquitin (K48-linkage specific) | rabbit | 1:1,1000* | D9D5/#8081 | Cell Signaling |
| V5-tag | mouse | 1:2,500 | SV5-Pk1/#R960-25 | Thermo Fisher Scientific |

For Western blot analysis, all antibodies were diluted in 1×TBS with 0.1% (v/v) Tween-20 and 0.02% (w/v) NaN_3_. *Antibody dilutions were supplemented with 5% (w/v) bovine serum albumin.
